# Enhancing secondary metabolite accumulation in medicinal plants to improve immunomodulatory activity

**DOI:** 10.3389/fphar.2026.1812475

**Published:** 2026-06-05

**Authors:** Phumzile Mkhize, Vivian Morafo, Zukile Mbita

**Affiliations:** Department of Biochemistry, Microbiology and Biotechnology, University of Limpopo, Sovenga, South Africa

**Keywords:** biostimulants, elicitors, immunoregulatory potential, increased accumulations of secondary metabolites, medicinal plants, metabolic synthesis, secondary metabolites

## Abstract

Despite progress in enhancing secondary metabolite accumulation to support plant growth and stress tolerance, relatively few studies have established direct links between these increases and enhanced immunoregulatory outcomes in humans. Bridging this gap is essential for advancing pharmacological applications and the development of effective plant-based immune therapeutics. Unlike previous reviews that primarily focus on metabolite enhancement strategies in isolation, this review proposes an integrative translational framework linking plant metabolic reprogramming, metabolite bioavailability, and downstream human immunomodulatory relevance. This review evaluates genetic, biochemical, and ecological strategies used to enhance secondary metabolite accumulation in medicinal plants and assesses their potential to translate into immunomodulatory effects. The available evidence indicates that while elicitor- and biostimulant-based approaches consistently enhance metabolite accumulation, their translation into clinically relevant outcomes is constrained by factors such as bioavailability, metabolic stability, and dose-dependent effects. The review further highlights key limitations and knowledge gaps, emphasising the need for integrative and translational research frameworks to link plant metabolic enhancement with human immune outcomes.

## 1 Introduction

**FIGURE 1:**
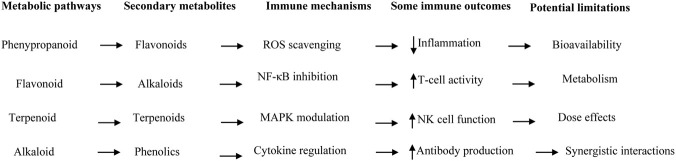
Integrated framework illustrating the proposed relationship between secondary metabolite biosynthetic pathways, metabolite accumulation in plants, and potential immunomodulatory effects in humans. Different secondary metabolite enhancement strategies trigger specific metabolic pathways in plants. These pathways lead to the biosynthesis of key secondary metabolites such as flavonoids, alkaloids, terpenoids, and phenolics. These metabolites potentially modulate immune mechanisms in humans, including reactive oxygen species (ROS) scavenging, nuclear factor kappa B (NF-κB) inhibition, mitogen-activated protein kinase (MAPK) signalling, and cytokine regulation, ultimately contributing to immune outcomes such as reduced inflammation and enhanced immune cell activity. However, the relationship may be influenced by key limitations, including bioavailability, metabolism, dose dependency, and synergistic interactions, which may affect the overall immunomodulatory efficacy.

**FIGURE 2:**
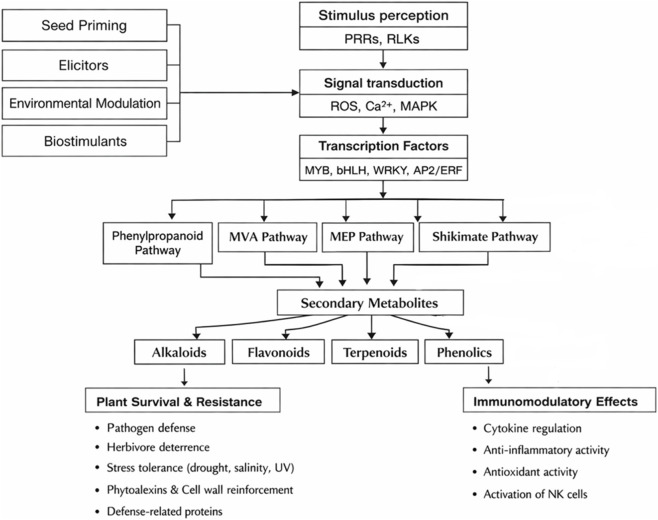
Mechanistic framework illustrating how elicitation strategies regulate secondary metabolite biosynthesis and their potential dual role in plant survival and immunomodulatory outcomes in medicinal plants. Elicitation approaches, including seed priming, elicitors, environmental modulation, and biostimulants, initiate stimulus perception through receptor-mediated recognition systems such as pattern recognition receptors (PRRs) and receptor-like kinases (RLKs). This triggers early signalling events, including reactive oxygen species (ROS) production, calcium (Ca^2+^) signalling, and mitogen-activated protein kinase (MAPK) cascades. These signalling pathways activate key transcription factors (e.g., MYB, bHLH, WRKY, AP2/ERF), which regulate gene expression associated with major biosynthetic pathways, including the phenylpropanoid, mevalonate (MVA), methylerythritol phosphate (MEP), and shikimate pathways. Enhanced metabolic flux through these pathways leads to increased accumulation of secondary metabolites such as alkaloids, flavonoids, terpenoids, and phenolic metabolites. These metabolites perform a dual function: (i) enhancing plant survival and resistance through mechanisms such as pathogen defence, herbivore deterrence, stress tolerance, induction of antimicrobial metabolites (phytoalexins and phytoanticipins), reinforcement of cell walls, and activation of defence-related proteins; and (ii) contributing to immunomodulatory effects in humans, including cytokine regulation, anti-inflammatory activity, antioxidant activity, and activation of natural killer (NK) cells. The figure highlights the hierarchical and causal relationships linking elicitation strategies to biochemical and physiological outcomes.

Medicinal plants are widely recognised for their capacity to produce a broad spectrum of secondary metabolites including flavonoids, alkaloids, terpenoids and phenolics that possess pharmacological and immunomodulatory properties (Kumar et al., 2021; Zhou X. et al., 2023). In addition, medicinal plants produce plant polysaccharides (Wang X.-F. et al., 2022). The metabolites and polysaccharides have been associated with significant immunomodulatory, antioxidant, anti-inflammatory, and metabolic regulatory properties (Achan et al., 2011; Tripathi and Mishra, 2015; Duarte and Takahashi, 2022; Wang X.-F. et al., 2022). A conceptual framework linking secondary metabolite accumulation to immunomodulatory outcomes in humans has been outlined in Figure 1. While later sections (Figure 2) provide a mechanistic representation of the underlying plant signalling and biosynthetic processes. These figures are important for highlighting the potential role of plant-derived secondary metabolites in supporting both plant biochemical processes and human immunological health.

At the cellular level, multiple studies conducted both *in vitro* and, to a lesser extent, *in vivo* have demonstrated that selected secondary metabolites interact with key immune targets, including cytokine signalling pathways, transcription factors such as NF-κB, and antioxidant response systems, thereby modulating both innate and adaptive immune responses (Sharma et al., 2017; Mahomoodally et al., 2022; Paço et al., 2022; Hooda et al., 2024; Sharma et al., 2024). For example, curcumin, a polyphenolic metabolite from *Curcuma longa* L*. (Zingiberaceae)*, has been reported to suppress NF-κB activation and downregulate pro-inflammatory cytokines (Pradanto et al., 2022). Further, quercetin, a widely distributed flavonoid, modulates immune responses through antioxidant activity and regulation of signalling pathways such as MAPK and NF-κB (Pradanto et al., 2022). However, despite these documented bioactivities, the translation of increased metabolite concentration into consistent and clinically relevant immunomodulatory outcomes remains insufficiently established.

**FIGURE 3:**
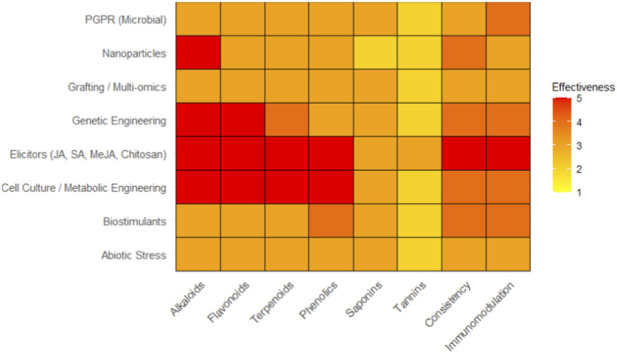
Presents a comparative heatmap summarising the relative effectiveness and consistency of different strategies used to enhance secondary metabolite accumulation in medicinal plants, alongside their associated immunomodulatory relevance. In this context, effectiveness refers to the magnitude of metabolite increase reported across studies, while consistency reflects the reproducibility and stability of these responses across different plant species, experimental conditions, and independent studies. The immunomodulatory relevance is inferred from the well-established biological activities of the associated metabolite classes, rather than being directly measured in the heatmap. PGPR, plant growth-promoting rhizobacteria; JA, jasmonic acid; SA, salicylic acid; MeJA, methyl jasmonate.

While extensive efforts have been dedicated to cataloguing the antimicrobial and therapeutic properties of various medicinal plants, comparatively less attention has been given to optimizing the accumulation of immunologically active secondary metabolites. Moreover, existing studies often assume a positive linear relationship between metabolite concentration and bioactivity. This is an assumption that may not hold due to factors such as bioavailability, synergistic interactions, and dose-dependent effects. Therefore, enhancing secondary metabolites biosynthesis, in conjunction with factors influencing bioavailability and functional efficacy is essential to fully harness this therapeutic potential. However, a clear conceptual framework linking plant metabolic enhancement to human immune outcomes is still lacking. In this context, we propose a conceptual framework in which (i) plant-level interventions (e.g., elicitation, priming, and microbial interactions) modulate biosynthetic pathways, (ii) this leads to quantitative and qualitative changes in metabolite profiles, and (iii) these changes influence specific molecular targets within the human immune system, subject to pharmacokinetic and pharmacodynamic constraints. To synthesise current evidence, a comparative heatmap (Figure 3) is presented to illustrate the relative effectiveness and consistency of different strategies used to enhance secondary metabolite accumulation in medicinal plants.

Advanced strategies have been investigated to increase the concentration and activity of secondary metabolites in medicinal plants, including biochemical engineering using cell suspension cultures to control metabolites production (Zhong, 2001). Investigations into the transcriptional regulation of biosynthetic pathways such as the phenylpropanoid, flavonoid, and terpenoid indole alkaloid pathways (Memelink et al., 2001). Genetic engineering approaches such as overexpressing biosynthetic and regulatory genes or silencing competing pathways using antisense technology, have proven effective in directing metabolic flux toward desired immune-relevant metabolites (Verpoorte and Memelink, 2002). Complementary metabolomics approaches have provided insight into metabolic profiles and pathway dynamics (Sumner et al., 2003). Functional genomics has facilitated the discovery of new genes responsive to elicitors and stress conditions that influence secondary metabolites biosynthesis (Goossens et al., 2003). Additionally, innovative tools such as grafting, integrated with multi-omics analysis, have uncovered spatial and temporal patterns of metabolite accumulation and translocation between rootstock and scion tissues (Regnault et al., 2016; Thomas and Frank, 2019; Dong et al., 2022).

Other promising strategies include the use of beneficial microbial consortia, such as specific bacterial combinations, to enhance plant metabolic activity and boost bioactive metabolite production (Lin et al., 2022). These plant growth-promoting rhizobacteria can improve the biosynthesis of phenolics and alkaloids by modulating hormonal signalling pathways linked to plant defence (Gowtham et al., 2022; Mashabela et al., 2022). Similarly, biostimulants including seaweed extracts, humic substances, and amino acids enhance plant resilience and secondary metabolism by mimicking stress signals or modulating phytohormones (Johnson et al., 2024; Mughunth et al., 2024). While these approaches effectively enhance metabolite accumulation *in planta*, their downstream relevance to human immunomodulation is often inferred rather than directly demonstrated (Figure 2; Table 1).

**TABLE 1:** Key classes of secondary metabolites and some strategies used to increase their accumulation in different plants, and their immunomodulatory actions.

Secondary metabolite	Immunomodulatory actions	References	Strategy category	Reported increase	References	Consistency across studies	Key synthesis/most effective strategy
Alkaloids	Enhance cytokine regulation, inhibit inflammatory enzymes, promote lymphocyte activity	Li et al. (2020); Zielińska et al. (2020); Plazas et al. (2025)	Elicitors (JA), Genetic (TF), Nanoparticles	25%–38% (JA); 3-fold (TF); very high with ZnO NPs	Al-Huqail and Ali, (2021); Sazegari et al. (2018); Alizadeh et al. (2025)	✓✓✓ High (Elicitors - JA)	Jasmonic acid is the most consistent inducer; genetic modification yields higher increases but is less scalable; nanoparticles show strong effects but limited field applicability
Flavonoids	Scavenge ROS, modulate NF-κB and MAPK pathways, enhance T-cell function	Hosseinzade et al. (2019); Behl et al. (2021); Zahra et al. (2024)	Light stress, Elicitors (MeJA), Temperature stress	20% (light); up to 2.7-fold (MeJA); variable (temperature)	Zhu et al. (2023); Wang et al. (2015); Alhaithloul et al. (2021)	✓✓ Moderate-High (Elicitors - MeJA)	MeJA elicitation is most effective and consistent; abiotic stress effects are variable and species-dependent
Terpenoids	Modulate macrophage and NK cell activity, anti-inflammatory and anticancer	Salminen et al. (2008); Paudel et al. (2023); Alarabei et al. (2024)	Elicitors (MeJA), Biotic stress, Herbivory/mechanical damage	5-fold (MeJA); 1.5–2.6-fold (Biotic stress); 15-fold (Herbivory/mechanical damage)	Martin et al. (2002); Kang et al. (2009); Opitz et al. (2008)	✓✓✓ High (Elicitors/Herbivory)	Jasmonates and herbivory-related stress provide the highest and most consistent induction across systems
Phenolic acids	Antioxidant, anti-inflammatory, reduce cytokine storms	Nadeem et al. (2019); Jia et al. (2023); Plazas et al. (2025)	Light (UV-B), Chemical elicitors, Oxidative stress	36% (UV-B); 35% (Oxidative stress); 1.3–2.2 (Chemical elicitors)	Lan et al. (2024); Wang W. et al. (2022); Takahashi et al. (2021)	✓✓ Moderate (Light + Chemical elicitors)	Combined light and chemical elicitors show consistent enhancement; responses vary with environmental conditions
Saponins	Stimulate antibody production, enhance dendritic cell function	Lucin et al. (2007); Shen et al. (2023)	Abiotic stress, Elicitors (SA, JA), Agronomic practices	6-fold (Abiotic stress); 2 (SA, JA); o 112% (Agronomic practices)	De Costa et al. (2013); Li et al. (2021)	✓✓ Moderate (Combined stress + elicitors)	Combined stress and elicitor treatments yield highest accumulation; field-based approaches show strong applicability
Tannins	Modulate gut immunity, anti-allergic and probiotic effects	Molino et al. (2022); Xu F. et al. (2023); Xu H. et al. (2023); Cosme et al. (2025)	Agronomic practices (harvesting)	Moderate increase (25–36 g/kg DW range)	Du Toit et al. (2020)	✓ Low (Agronomic practices)	Limited studies: agronomic practices influence accumulation but lack consistency and mechanistic understanding

Consistency across studies reflects the reproducibility of the most effective or dominant strategy identified within each category, rather than all listed strategies equally. JA, jasmonic acid; MeJA, methyl jasmonate; SA, salicylic acid; UV-B, ultraviolet-B radiation; ROS, reactive oxygen species; NF-κB, nuclear factor kappa B; MAPK, mitogen-activated protein kinase; NK, natural killer; DW, dry weight.

Elicitor treatments using molecules like jasmonic acid (JA), salicylic acid (SA) and chitosan stimulate secondary metabolite biosynthesis through defence and immune-like signalling pathways (Kandoudi and Németh-Zámboriné, 2022). These elicitors are widely recognised for their role in triggering plant defence signalling cascades that result in increased production of bioactive secondary metabolites (Lu, 2009; Ruiz-García and Gómez-Plaza, 2013). This is achieved through responses such as changes in cellular redox balance, ion channel activity, and kinase signalling (Ninkuu et al., 2021; Shah et al., 2023; Shah and Gupta, 2023). Such events modulate the expression of key genes involved in secondary metabolite biosynthesis and pathogenesis-related responses (Shilpa et al., 2025).

Environmental conditions such as light quality, temperature shifts, drought, and soil nutrient dynamics also play critical roles in regulating plant stress responses, which are tightly linked to secondary metabolite production (Pant et al., 2021; Mkhize et al., 2023a; Qaderi et al., 2023). Across studies, elicitor-based strategies, particularly jasmonates, consistently resulted in the highest and most reproducible increases in secondary metabolite accumulation across different plant systems. While genetic approaches achieved greater fold increases, however, their practical applicability remains limited. Abiotic stress treatments showed moderate but variable effects depending on species and environmental conditions. Overall, elicitor-based approaches emerge as the most reliable and scalable strategy for enhancing secondary metabolite production (Table 1).

Importantly, although these plant responses are well characterised, their implications for human immune modulation depend on additional factors such as metabolite stability, absorption, metabolism, and interaction with human immune cells. These are not consistently addressed in current plant-focused studies. This review is therefore guided by the hypothesis that targeted enhancement of secondary metabolite accumulation in medicinal plants can improve immunomodulatory potential. However, this effect is not determined by concentration alone and depends on metabolite-specific bioactivity, bioavailability, and interactions among metabolites. Accordingly, the objective of this review is to critically evaluate current strategies for enhancing secondary metabolite accumulation in medicinal plants and to assess the extent to which such enhancements can plausibly translate into immunomodulatory effects, while highlighting existing limitations and research gaps. This systems-level interpretation represents a key conceptual advancement over previous descriptive reviews by integrating biochemical enhancement with translational immunopharmacological relevance.

## 2 Materials and methods

### 2.1 Literature search strategy

This review adopted a structured literature search approach guided by the Preferred Reporting Items for Systematic Reviews and Meta-Analyses (PRISMA) recommendations to improve transparency, reproducibility, and comprehensive literature coverage. A structured literature search was performed to identify studies focusing on strategies for enhancing secondary metabolite accumulation in medicinal plants and their associated immunomodulatory potential. Electronic databases including PubMed, ScienceDirect, Google Scholar and Scopus, were systematically searched for relevant literature published between 2000 and 2025. Additional articles were identified through manual screening of reference lists from eligible studies and relevant review articles.

The search strategy employed combinations of keywords and Boolean operators, including:

- medicinal plants AND secondary metabolites
- secondary metabolite biosynthesis AND immunomodulation
- elicitation OR elicitors AND phytochemicals
- biostimulants AND medicinal plants
- plant secondary metabolites AND immune response
- metabolic engineering AND bioactive metabolites
- CRISPR/Cas AND secondary metabolites
- environmental stress AND phytochemical accumulation
- plant metabolomics AND medicinal plants
- bioavailability AND polyphenols
- transcription factors AND specialized metabolism

Search terms were adapted where necessary to suit the indexing systems of different databases. Boolean operators (AND, OR), truncation symbols, and Medical Subject Headings (MeSH) terms were used where applicable to maximise retrieval efficiency and minimise omission of relevant studies.

### 2.2 Inclusion and exclusion criteria

Studies were considered eligible for inclusion if they met the following criteria:

- peer-reviewed original research articles, systematic reviews, or review papers published in English
- studies focusing on medicinal or aromatic plants and the biosynthesis, enhancement, regulation, or accumulation of secondary metabolites
- studies investigating enhancement strategies including elicitation, biostimulants, phytohormones, environmental manipulation, abiotic stress, metabolic engineering, genome editing, molecular breeding, hydroponics, tissue culture, or microbial inoculation
- studies reporting quantitative or qualitative data on phytochemical accumulation, metabolite profiling, antioxidant activity, anti-inflammatory activity, or immunomodulatory effects
- studies evaluating the therapeutic relevance, bioavailability, pharmacological activity, or mechanistic pathways associated with plant secondary metabolites.

Studies were excluded if they:

- were non-peer-reviewed publications, conference abstracts, theses, or duplicate records
- lacked sufficient methodological or phytochemical information
- focused on unrelated agricultural or pharmaceutical topics without relevance to secondary metabolite enhancement or immunomodulation
- did not provide scientifically interpretable biological, biochemical, or metabolomic outcomes.

### 2.3 Study selection process

The study selection process was conducted guided by the PRISMA recommendations. Screening was performed in three sequential stages:

- Title screening
- Abstract screening

Studies that did not satisfy the inclusion criteria during title or abstract screening were excluded. Full-text articles were then critically evaluated for methodological relevance, scientific quality, and alignment with the objectives of the review. A transparent screening and selection process was followed to ensure inclusion of relevant and high-quality studies.

### 2.4 Data extraction and qualitative synthesis

Relevant data were systematically extracted from eligible studies using a standardised data extraction framework. Extracted information included:

- plant species and family
- metabolite classes investigated
- enhancement strategy employed
- experimental conditions
- analytical and metabolomic techniques used
- phytochemical outcomes
- reported antioxidant, anti-inflammatory, or immunomodulatory activities
- proposed molecular or biochemical mechanisms.

A qualitative synthesis approach was employed to compare findings across studies and identify recurring trends, mechanistic insights, and effective enhancement strategies. Emphasis was placed on integrating evidence relating to elicitor signalling, transcriptional regulation, metabolic engineering, environmental modulation, and biostimulant-mediated metabolic reprogramming. Tables and figures were developed to summarise enhancement strategies, metabolite classes, associated therapeutic activities, and mechanistic pathways contributing to immunomodulatory efficacy.

### 2.5 Taxonomic validation of plant species

All plant species included in this review were taxonomically validated using authoritative botanical databases. Scientific names were standardized to include full binomial nomenclature, including author citations, family classification and common names to ensure taxonomic accuracy and consistency throughout the manuscript.

### 2.6 Quality assessment and evidence reliability

To improve scientific rigour and minimise bias, studies included in this review were critically evaluated based on methodological clarity, experimental reproducibility, analytical robustness, and relevance to the review objectives. Consideration was given to studies employing validated analytical approaches such as Liquid Chromatography-Mass Spectrometry and Gas Chromatography-Mass Spectrometry, metabolomics platforms, transcriptomics, and molecular characterisation techniques.

## 3 Improving immunomodulatory potential in medicinal plants through biostimulant and elicitor-induced secondary metabolite enhancement

### 3.1 Biostimulants

Biostimulants are natural or synthetic substances and/or microorganisms that enhance plant growth, nutrient uptake, and stress tolerance without acting as direct fertilizers or pesticides (Bulgari et al., 2015; Bulgari et al., 2019; Mrid et al., 2021; Franzoni et al., 2022). They can be applied through foliar spraying, soil or fertigation treatments, seed priming, or transplant/root dipping. Each application method targets specific outcomes such as improved germination, root development, or stress resilience. Foliar application enables rapid uptake, whereas soil and fertigation applications promote root activity and beneficial microbial interactions. However, the effectiveness of biostimulants is highly context-dependent and can vary significantly depending on plant species, genotype, developmental stage, environmental conditions, and application method. For example, the same biostimulant may enhance phenolic accumulation under abiotic stress conditions but show limited or inconsistent effects under optimal growth conditions (Ali et al., 2021). Variability in extract composition, particularly in seaweed-derived products, further contributes to inconsistent outcomes due to differences in species origin, harvesting time, and extraction techniques (Ali et al., 2021). In addition, dosage and application frequency can influence responses, with excessive concentrations sometimes leading to metabolic imbalances or negligible gains in secondary metabolite production (Santini et al., 2021; Patanè et al., 2025). These limitations highlight the need for standardisation, optimisation, and context-specific application strategies when evaluating biostimulant efficacy.

Seaweed extracts are among the most widely used biostimulants. They are rich in phytohormones (auxins, cytokinins), polysaccharides, and micronutrients (Ali et al., 2021). Seaweed extracts promote the synthesis of phenolics, flavonoids, and terpenoids and enhance antioxidant activity and stress tolerance (Zulfiqar et al., 2020; Ali et al., 2021). In Table 2, several studies are presented that have utilized seaweed extracts to enhance secondary metabolite accumulation in different crops. The biochemical mechanisms underlying these effects are largely associated with the activation of key metabolic pathways, such as the phenylpropanoid, flavonoid, and terpenoid biosynthetic pathways. This activation promotes the synthesis of various classes of secondary metabolites. Information on their chemical structures, stability, and immunomodulatory effects is also summarized in Table 2. Further, seaweed extracts can enhance plant polysaccharide accumulation by modulating metabolic and signalling pathways involved in carbohydrate biosynthesis and secondary metabolite production (Rachidi et al., 2020).

**TABLE 2:** Summary of studies reporting the use of seaweed extracts to enhance secondary metabolite accumulation in various crops, highlighting activated pathways, metabolite types and representative metabolites enhanced in each of the study, chemical structures, stability, and immunomodulatory effects.

Crop	Seaweed extract source	Activated metabolic pathway(s)	Enhanced secondary metabolite(s)	Representative metabolite(s)	Chemical structure and stability	Reported immunomodulatory effects	Experimental robustness/reproducibility/statistical significance	Meta-level interpretation/effectiveness
*Vigna unguiculata* (L.) Walp. (Fabaceae) (cowpea) and *Zea mays* L. (Poaceae) (maize) (Hussein et al., 2021)	Egyptian seaweeds (*Ulva fasciata*, *Cystoseira compressa*, and *Laurencia obtusa*) extract (Hussein et al., 2021)	Phenylpropanoid pathway (Shende et al., 2024)	Phenolics (Hussein et al., 2021)	Polyphenol (Hussein et al., 2021)	Polyphenols are metabolites with multiple aromatic rings bearing hydroxyl groups, and their structural complexity strongly influences their stability, solubility, and bioavailability (Panche et al., 2016)	Polyphenols biological activities, including antioxidant, anti-inflammatory, antitumor, cardioprotective, and neuroprotective effects (Dong et al., 2024)	Significant increase in phenolic content reported (p < 0.05); replicated across multiple treatments but limited independent validation	Strong evidence for phenylpropanoid activation; consistent increase in phenolics across crops suggests high reproducibility
*Capsicum annuum* L*.* (Solanaceae*)* (sweet pepper) (Rajendran et al., 2021)	*Ascophyllum nodosum* extract (Rajendran et al., 2021)	Phenylpropanoid and flavonoid pathways (Shende et al., 2024)	Phenolics and flavonoids (Rajendran et al., 2021)	Quercetin and catechin (Rajendran et al., 2021)	Quercetin and catechin are polyphenolic metabolites with multiple hydroxyl groups. However, they differ in the specific positioning of their hydroxyl groups: quercetin, a flavanol, contains five hydroxyl groups, whereas catechin, a flavan-3-ol, has a distinct substitution pattern. They are moderately heat-stable but sensitive to oxidation and UV (Malaguti et al., 2013; Ebrahimi et al., 2024)	Potent antioxidant and anti-inflammatory effects (through suppressing pro-inflammatory cytokines like IL-6 and TNF-α, enhancing natural killer cell activity, and modulating immune pathways like NF-κB.; promotes macrophage activation and cytokine modulation (Li et al., 2019; Ebrahimi et al., 2024)	Reported statistically significant increases (p < 0.05); moderate sample size; good reproducibility within experimental conditions	Highly consistent activation of flavonoid pathway; strong immunomodulatory relevance across studies
*Achillea millefolium* L. (Asteraceae) (yarrow*)* (Shafie et al., 2021)	Seaweed extract + Amino acids (Shafie et al., 2021)	Shikimate-phenylpropanoid pathway (Falcone Ferreyra et al., 2012)	Phenols and flavonoids (Shafie et al., 2021)	Not specified	Phenols contain a hydroxyl group bonded to an aromatic ring, whereas flavonoids are a subclass of polyphenols with a three-ring. Flavonoids are more susceptible to auto-oxidation, while phenols are relatively stable, although stability decreases with additional hydroxyl groups or specific oxidation states (Dai and Mumper, 2010; Pasquet et al., 2020)	Phenols and flavonoids modulate immunity by reducing oxidative stress and inflammation, regulating cytokine production, and supporting immune homeostasis, with potential therapeutic effects in autoimmune and inflammatory conditions (Azab et al., 2016; Han et al., 2021)	Significant increases reported (p < 0.05); limited replication across independent studies	Combination treatments (seaweed + amino acids) show enhanced but less consistently validated responses
*Curcuma longa* L. (Zingiberaceae) (turmeric) (Pradanto et al., 2022)	*Sargassum muticum* extract	Terpenoid and phenylpropanoid pathway (Shende et al., 2024)	Curcuminoids (Pradanto et al., 2022)	Curcumin, demethoxycurcumin (Pradanto et al., 2022)	Curcumin, which contains two methoxy groups, is less stable and more prone to degradation in neutral or basic aqueous conditions, whereas demethoxycurcumin, lacking methoxy groups, exhibits greater stability (Hatamipour et al., 2019)	Curcumin and demethoxycurcumin modulate immunity by enhancing pathogen defence and suppressing excessive inflammation, including reduce excessive activation of inflammation and allergy, can suppress intracellular NF-κB, MAPKs, JAKs/STATs, β-catenin, and the Notch-1 pathway (Momtazi-Borojeni et al., 2018; Kahkhaie et al., 2019)	Significant upregulation (p < 0.05); controlled experimental setup but limited field validation	Terpenoid-phenylpropanoid crosstalk suggests robust pathway activation with strong pharmacological relevance
*Ocimum basilicum* L. (Lamiaceae) (basil) (Lam et al., 2025)	*Kappaphycus alvarezii* extract (Lam et al., 2025)	Phenylpropanoid pathway (Shende et al., 2024)	Phenolic acids and terpenoids (Lam et al., 2025)	Rosmarinic acid, eugenol (Lam et al., 2025)	Rosmarinic acid is a polyphenol with phenolic and carboxylic groups, moderately stable at 37 °C and pH 7.5 but degrades in acidic conditions. Eugenol is a volatile aromatic phenol with hydroxyl, methoxy, and allyl groups, relatively stable but prone to oxidation and best stored in glass or tin (Islamčević Razboršek, 2011; Charoensin and Dansakda, 2023)	Rosmarinic acid reduces allergic reactions, inhibits inflammatory pathways such as NF-κB, and can trigger apoptosis in specific immune cells, whereas eugenol suppresses pro-inflammatory cytokines and stimulates macrophage and natural killer cell activity (Luo et al., 2020; Noor et al., 2022)	Statistically significant increases reported (p < 0.05); moderate reproducibility across replicates	Strong and consistent phenylpropanoid activation with reliable immunomodulatory effects
*Glycine max* (L.) Merr. (Fabaceae) (soybean) (Shukla et al., 2017)	*Ascophyllum nodosum* extract (Shukla et al., 2017)	Isoflavonoid biosynthetic pathway (Shende et al., 2024)	Isoflavones (Shukla et al., 2017)	Genistein, daidzein (Shukla et al., 2017)	Genistein contains three hydroxyl groups, whereas daidzein has two, with the primary structural difference being an additional hydroxyl at the 5-position on genistein’s isoflavone backbone. Both metabolites are relatively stable, with genistein maintaining stability in formulated products for up to 2 years, while daidzein is slightly less stable, which can affect its water solubility and bioavailability (Ungar et al., 2003; Intharuksa et al., 2025)	daidzein can enhance lymphocyte activation, while genistein acts as an immunosuppressant by inhibiting immune cell signalling and enhancing certain immune responses like cytotoxic T-cell and natural killer cell activity (Smith and Dilger, 2018)	Significant increases (p < 0.05); well-documented but mostly greenhouse/controlled studies	Isoflavonoid pathway consistently activated; strong evidence for reproducibility and functional immune effects

Overall synthesis: dominant pathways = phenylpropanoid-, flavonoid-, terpenoid-, and isoflavonoid-associated pathways were the most consistently activated metabolic networks across seaweed-based biostimulant systems; major trend = seaweed-derived biostimulants generally enhanced secondary metabolite accumulation across diverse plant species, indicating conserved stress-responsive metabolic activation; reproducibility limitation = responses varied according to species, genotype, environmental conditions, dosage, and application strategy, limiting consistency and scalability across production systems; main translational constraint = increased metabolite accumulation did not consistently translate into validated immunomodulatory efficacy due to limitations associated with metabolite stability, bioavailability, and functional immune validation; key knowledge gap = most studies were conducted under controlled experimental conditions, with limited field-scale, long-term, and clinically relevant validation; conceptual insight = unlike previous descriptive approaches focused primarily on metabolite enhancement, this table integrates metabolic pathway activation, translational limitations, and downstream immunomodulatory relevance to provide a more systems-level framework for evaluating therapeutic potential.

At the mechanistic level, seaweed-derived biostimulant-induced enhancement of secondary metabolism is not uniform, but rather involves the selective activation of specific signalling pathways and biosynthetic routes. Instead of broadly stimulating overall metabolism, these biostimulants differentially regulate pathway activity depending on the nature of the stimulus and plant context, resulting in the targeted accumulation of specific metabolite classes. These responses are mediated by key signalling molecules, including jasmonic acid and salicylic acid, which function as central regulators linking external stimuli to metabolic reprogramming (Sudatti et al., 2020; Ali et al., 2021). A more detailed discussion of pathway-level regulation and transcriptional control is provided in Section 3.2.

### 3.2 Metabolic pathways

The table (Table 2) reflects on the different activated pathways. Plants possess a highly coordinated metabolic regulatory system that allows selective activation of secondary metabolite pathways depending on specific signals, elicitors, or stresses. This regulation operates across multiple hierarchical levels, including signal perception, transcriptional control, enzymatic activity, and metabolic flux redistribution. Distinct stimuli, such as pathogen attack, light stress, elicitor application, or biostimulants like seaweed extracts, humic acids, and fulvic acids, trigger signalling molecules such as jasmonic acid, salicylic acid, or mitogen-activated protein kinase cascades, each leading to distinct metabolic outputs depending on pathway activation and cellular context (Hounsome et al., 2008; Boutahiri et al., 2024).

Beyond pathway activation, the extent of secondary metabolite accumulation is strongly governed by metabolic flux distribution, rate-limiting enzymatic steps, and pathway bottlenecks. In many biosynthetic pathways, key enzymes such as phenylalanine ammonia-lyase in the phenylpropanoid pathway or 3-hydroxy-3-methylglutaryl coenzyme A reductase in terpenoid biosynthesis function as major control points regulating carbon flux (Parađiković et al., 2019; Li et al., 2020). Even when signalling pathways are activated, limitations in precursor availability, feedback inhibition, and competition between parallel pathways can constrain metabolite accumulation. For instance, carbon partitioning between primary metabolism (growth and maintenance) and secondary metabolism often results in trade-offs, particularly under non-stress or resource-limited conditions (Sudatti et al., 2020; Ali et al., 2021). Therefore, enhancing secondary metabolite production requires not only pathway induction but also the alleviation of metabolic bottlenecks and optimisation of flux toward target biosynthetic routes. For example, salicylic acid predominantly activates phenylpropanoid and flavonoid synthesis, increasing phenolic acids, flavonoids, and tannins (Parađiković et al., 2019; Li et al., 2020). Moreover, biostimulants like seaweed extracts specifically stimulate jasmonic acid and salicylic acid signalling, promoting both phenolic and terpenoid accumulation depending on the formulation and application method (Sudatti et al., 2020; Ali et al., 2021).

Master regulators, such as MYC2 in jasmonate signalling or NPR1 in salicylic acid signalling, function at the top of regulatory cascades, integrating environmental and hormonal cues (Peñuelas et al., 2019; Zeng et al., 2025). These central regulators control downstream transcription factors including WRKY, MYB, and basic helix-loop-helix proteins, which in turn regulate structural genes encoding biosynthetic enzymes (Schluttenhofer and Yuan, 2015). Cross-talk between signalling pathways, such as antagonistic or synergistic interactions between jasmonic acid and salicylic acid, further refines pathway specificity and metabolic outcomes (Schluttenhofer and Yuan, 2015). In addition, transcription factor complexes and co-regulators enable system-level coordination, ensuring the synchronized expression of entire biosynthetic gene networks rather than single enzymatic steps. This hierarchical and integrative control ultimately determines pathway prioritisation, metabolic flux direction, and the efficiency of secondary metabolite accumulation (Liu et al., 2023; Huang et al., 2024; Zeng et al., 2025). For example, the jasmonic acid-coronatine-insensitive 1–MYC2 pathway primarily governs terpenoid and alkaloid biosynthesis. In this case, jasmonate signalling activates transcription factors such as MYC2, octadecanoid-responsive Catharanthus roseus AP2/ERF-domain (ORCA), and basic helix-loop-helix (bHLH) proteins. These control enzymes like 1-deoxy-D-xylulose-5-phosphate synthase, 3-hydroxy-3-methylglutaryl coenzyme A reductase, and strictosidine synthase (Peñuelas et al., 2019; Zeng et al., 2025). These signalling cascades ultimately target key regulatory elements such as transcription factors, rate-limiting enzymes, metabolite transporters, and signal transducers, which together coordinate the diversion of carbon and energy toward specific secondary metabolite synthesis (Liu et al., 2023; Huang et al., 2024; Zeng et al., 2025). This regulatory flexibility is critical for research aiming to enhance secondary metabolites in medicinal plants. Understanding and manipulating which signalling pathways are activated, which transcription factors are expressed, and how metabolic flux is allocated enables precise enhancement of pharmacologically active metabolites, providing a biotechnological handle to optimize medicinal plant potency and functional diversity.

### 3.3 Chemical structures and stability of secondary metabolites

Information regarding chemical structures has been incorporated into Table 2. Examining the chemical structures and stability of secondary metabolites is essential for interpreting the functional implications of their enhanced accumulation. The structural complexity of secondary metabolites not only influences their stability and solubility, but also directly determines their bioavailability, pharmacokinetics and ultimately their immunomodulatory efficacy (Manach et al., 2004; Manach et al., 2005; Junio et al., 2011). Similarly, structural characteristics, including monosaccharide composition, glycosidic linkages and branching patterns affect functional implications of plant polysaccharides (Wang X.-F. et al., 2022). In addition, molecular size, polarity, and functional group composition govern intestinal permeability, metabolic transformation, and systemic circulation time, which are critical determinants of biological activity *in vivo*. Smaller molecules (<500 Da) exhibit favourable membrane permeability and can readily diffuse across lipid bilayers, resulting in efficient intestinal absorption and systemic availability (Samsonowicz et al., 2017). However, a substantial proportion of plant secondary metabolites, including glycosylated flavonoids, saponins, and tannins, exceed this threshold and display reduced passive permeability (Lewandowska et al., 2016). These larger metabolites often require enzymatic hydrolysis, microbial biotransformation, or active transport mechanisms to become bioavailable. Moreover, increased molecular size is frequently associated with reduced solubility and enhanced susceptibility to metabolic modification, which may limit systemic exposure but can still support local or indirect immunomodulatory effects.

To address this, emerging evidence suggests that microbial and hepatic biotransformation may significantly influence the pharmacological activity of medicinal plant metabolites. For example, Hu et al. (2023) demonstrated that microbial and hepatic transformation of astragaloside IV generated metabolites with substantially improved bioavailability and blood-brain barrier permeability compared to the parent metabolites. Importantly, these transformed metabolites exhibited enhanced biological activity through modulation of inflammatory and immune-related targets, including TNF, CSF1R, CDC42, and PTK2, resulting in suppression of microglial proliferation, migration, and inflammatory cytokine secretion (Hu et al., 2023). These findings highlight that improvements in metabolite accumulation alone may not fully determine therapeutic efficacy unless factors such as biotransformation, absorption, pharmacokinetics, and metabolite-target interactions are also considered. Consequently, future strategies aimed at enhancing immunomodulatory metabolites in medicinal plants should integrate phytochemical enhancement with pharmacokinetic and mechanistic validation approaches to improve translational applicability.

Labile functional groups, including esters, glycosides, and epoxides, are highly susceptible to enzymatic and non-enzymatic degradation, such as hydrolysis in the gastrointestinal tract, oxidation during storage, or biotransformation in the liver. These processes may significantly reduce bioactive metabolite concentrations or generate metabolites with altered or reduced immunomodulatory activity (Spigno et al., 2007; Carrera et al., 2012; Lee et al., 2024).

Hydrophobic metabolites, such as many terpenoids and curcuminoids, often exhibit low aqueous solubility, rapid metabolism, and poor systemic bioavailability, which limits their therapeutic efficacy despite high accumulation in plant tissues. For example, curcumin is well known for its rapid degradation and limited absorption, resulting in low plasma concentrations unless stabilised or formulated appropriately (Gupta et al., 2023). These considerations highlight a critical limitation: enhanced metabolite accumulation *in planta* may not necessarily translate into improved therapeutic outcomes, unless stability, delivery, and pharmacokinetic constraints are addressed. Therefore, integrating structural chemistry with pharmacokinetic behaviour is essential to accurately predict the functional immunomodulatory potential of metabolites under physiological conditions.

Building on this, hydroxyl groups in polyphenols such as flavonoids (characterized by a C_6_ – C_3_ – C_6_ skeletal structure) enhance radical scavenging and immunomodulatory signalling, but can reduce membrane permeability and metabolic stability, thereby limiting systemic bioavailability (Williamson, 2002; Spencer et al., 2004; Şengül et al., 2025). Structural modifications such as glycosylation improve solubility but may require enzymatic hydrolysis prior to absorption. While advanced formulation strategies, including liposomes, phytosomes, nanoemulsions, and polymer-based nanoparticles, have been shown to significantly enhance stability, permeability, and targeted delivery (Rein et al., 2013; Olfati et al., 2025). Similarly, heteroatoms such as nitrogen (alkaloids) and sulfur (glucosinolates) influence molecular reactivity, metabolic conversion, and interaction with immune-related targets. While the lipophilic-hydrophilic balance governs membrane transport and tissue distribution (Rein et al., 2013). Understanding these structure-function relationships enables the design of targeted delivery systems and stabilisation strategies, which are essential for maintaining metabolite integrity during storage, processing, and *in vivo* application. Likewise, the public can apply this knowledge through dietary or preparation strategies, including combining plant products with natural bioenhancers (e.g., piperine or healthy oils) to improve the bioavailability and activity of secondary metabolites, particularly where biochemical or environmental interventions have increased their accumulation.

A major challenge in translating plant-derived secondary metabolites into therapeutic applications is their chemical instability and degradation during processing, storage, and physiological exposure. Factors such as light, temperature, oxygen, and pH can induce degradation pathways including oxidation, isomerisation, and hydrolysis. For example, flavonoids are prone to oxidative degradation, while terpenoids may undergo volatilisation or structural rearrangement (Kumar and Pandey, 2013; Speisky et al., 2022). To overcome these limitations, encapsulation and controlled delivery systems have emerged as critical strategies. Techniques such as nanoencapsulation, solid lipid nanoparticles, cyclodextrin inclusion complexes, and biodegradable polymer carriers can protect metabolites from degradation, enhance bioavailability, and enable sustained release (Hao et al., 2026). In this context, Zeng et al. (2022) emphasised that the combination of nanomedicine with traditional Chinese medicine (TCM) provides a strategic platform for enhancing the delivery and bioactivity of metabolites. Similarly, Li K. et al. (2026) highlighted that metal ion-mediated delivery systems can enhance the transport and functional modulation of metabolites through mechanisms such as biomimetic mineralisation, metal-organic frameworks, self-polymerisation, and *in situ* mineralising hydrogels. These platforms improve transport across biological barriers, including cellular membranes, oral barriers, and the blood-brain barrier, while also enabling controlled release and targeted therapeutic activity. Importantly, the authors noted that such systems may improve metabolite stability and therapeutic efficacy but also require careful consideration of ion-associated toxicity, premature cargo release, and biosafety. These advanced formulation and delivery approaches help address challenges such as poor bioavailability, variability in composition, and inconsistent therapeutic outcomes.

Recent advances in targeted drug delivery further demonstrate the importance of improving the pharmacokinetic stability and tissue-specific delivery of bioactive metabolites. Zheng et al. (2025) developed an Fc-mediated small molecule-drug conjugate delivery platform that significantly prolonged circulating half-life and enhanced therapeutic efficacy through receptor-targeted delivery and FcRn-mediated recycling. The study highlighted that limited *in vivo* stability and short circulation time remain major barriers to the clinical translation of small bioactive metabolites, reducing tissue exposure and therapeutic responsiveness. Importantly, prolonged delivery systems improved tumour targeting and enhanced biological activity while reducing dosing frequency. Although developed primarily for anticancer therapeutics, these findings provide important translational insights for plant-derived secondary metabolites, many of which exhibit poor bioavailability, rapid degradation, and limited systemic persistence.

In addition to chemical stability and delivery-related limitations, the successful clinical translation of plant-derived metabolites is influenced by regulatory, manufacturing, and acceptance-related challenges across both traditional and modern biomedical systems. A key argument presented by Zeng et al. (2022) is that both nanomedicine and traditional medicine face shared challenges related to regulatory approval, manufacturing standardisation, toxicity evaluation, and public acceptance. The authors emphasise that addressing these challenges through harmonised regulatory frameworks and improved scientific validation is essential for successful clinical translation. This supports the need for rigorous, evidence-based approaches when evaluating plant-derived metabolites and their immunomodulatory claims, particularly in contexts where scalability and reproducibility remain limiting factors.

### 3.4 Diversity of biostimulants and their mechanistic role in secondary metabolite biosynthesis

In addition to the crops listed in Table 2, seaweed biostimulants enhance secondary metabolite accumulation in several other medicinal and aromatic plants, including *Achillea millefolium* L. (Asteraceae) (yarrow), *Abelmoschus esculentus* L. (Malvaceae) (okra), *Vicia faba* L. (Fabaceae) (fava bean), *Echinacea purpurea* L. (Asteraceae) (purple coneflower), *Zingiber officinale Roscoe* (Zingiberaceae) (ginger), *Camellia sinensis* (L.) Kuntze (Theaceae) (tea plant), and *Psidium guajava* L. (Myrtaceae) (guava) (Shafie et al., 2021; Aremu et al., 2022; Shahrajabian and Sun, 2022; El Boukhari et al., 2023; Çakmak and Uzuner, 2023). For example, *Camellia sinensis* (L.) Kuntze, Theaceae (green tea) contains potent catechins such as epigallocatechin-3-gallate and epicatechin-3-gallate, polyphenolic metabolites with strong antioxidant, anti-inflammatory and anticancer activities. These also have immunomodulatory activities including inhibition of nuclear factor kappa B signalling, modulation of Toll-like receptor pathways, and enhancement of macrophage and natural killer cell activity (Watson et al., 2005; Youn et al., 2006; Braun et al., 2009). This highlights the potential of seaweed biostimulants, and future studies applying these extracts to medicinal plants could further enhance bioactive metabolite accumulation and elucidate their mechanisms, not only for agronomic outcomes but also for the development of novel therapeutic applications.

Other categories of biostimulants include humic and fulvic acids (1), which enhance nutrient uptake, root development, and enzymatic activities linked to secondary metabolite biosynthesis, e.g. phenylalanine ammonia-lyase (Quijia et al., 2024). Amino acids and peptides (2), act as signalling molecules and precursors for secondary metabolites, improving plant growth, metabolism, and stress responses, which often trigger secondary metabolite production (Bina and Rahimi, 2017; Sun et al., 2024). Protein hydrolysates (3), derived from enzymatic, chemical, or thermal hydrolysis of organic residues. These are rich in polypeptides and free amino acids such as glutamate, glutamine, proline, and glycine (Wieczorek et al., 2023; Pipiak et al., 2024; Famielec, 2015; Gendaszewska et al., 2021). They stimulate carbon and nitrogen metabolism and enhance the activity of enzymes producing flavonoids and phenolics (Colla et al., 2017). Amino acids (4) are readily absorbed through roots, leaves, or as seed treatments, influencing nitrogen metabolism and improving productivity (Sun et al., 2024). Beneficial microorganisms (5), including plant growth-promoting rhizobacteria and mycorrhizae, enhance nutrient uptake, stimulate root growth, induce systemic resistance, and promote secondary metabolite accumulation by modulating hormonal and signalling pathways (Rouphael et al., 2015; Rouphael and Colla, 2018; Rouphael and Colla, 2020). Microbial inoculants (6) can upregulate growth and defence-related secondary metabolism, increasing plant resilience to environmental stresses. Finally, chitosan and oligosaccharides (7) act as biostimulants by mimicking pathogen attack to activate defence signalling and stimulate phytoalexin and pathogenesis-related metabolite synthesis (Hazekawa et al., 2017). Collectively, these biostimulants stimulate secondary metabolite biosynthesis through diverse yet synergistic mechanisms, offering significant agronomic outcomes while also providing a promising avenue for enhancing the immunomodulatory potential of medicinal plants through targeted metabolite enrichment. Among these approaches, the combined regulation of phytohormonal signalling and nutrient availability has emerged as a particularly effective strategy for redirecting metabolic pathways toward enhanced biosynthesis of high-value secondary metabolites.

Supporting this concept, Li J. et al. (2026) demonstrated that the combined application of indole-3-acetic acid (IAA) and high nitrogen significantly enhanced lutein accumulation in *Chlorella protothecoides* (Chlorellaceae) (green alga) through redirection of carbon flux and modulation of carotenoid biosynthetic genes, including *CRTISO*, *LCYB*, *ZEP*, and *VDE*. The study further showed that fed-batch heterotrophic cultivation enhanced tricarboxylic acid cycle activity and promoted carotenoid flux, resulting in substantial increases in lutein productivity. These findings highlight the importance of integrating phytohormonal regulation with nutrient optimisation to improve the accumulation of high-value bioactive metabolites. Such approaches may provide scalable and cost-effective strategies for enhancing the production of secondary metabolites with potential antioxidant and immunomodulatory applications.

### 3.5 Biostimulant-induced metabolite enhancement: evidence, extraction strategies, and translational implications

Table 3 gives specific attention to the extraction methods as there is not a single technique that is accepted as the standard technique for extracting bioactive chemicals from plants. Conventional techniques such as maceration and Soxhlet extraction remain widely used due to their simplicity and low technological requirements. However, they are often associated with long extraction times, high solvent consumption, low selectivity, and increased risk of thermal and oxidative degradation of sensitive metabolites. These limitations reduce extraction efficiency and may compromise the integrity of bioactive metabolites. In contrast, advanced extraction technologies, including ultrasound-assisted extraction, microwave-assisted extraction, supercritical fluid extraction, and enzyme-assisted extraction, have been developed to overcome these limitations. These approaches offer enhanced extraction efficiency, reduced processing time, lower solvent usage, and improved preservation of thermolabile metabolites, thereby maintaining the structural integrity and biological activity of secondary metabolites (Bitwell et al., 2023; Ghenabzia et al., 2023).

**TABLE 3:** Enhancements of secondary metabolite accumulation in medicinal using humic and fulvic, microbial-based, amino acid, and protein hydrolysate biostimulants across various crops.

Crop	Common immunomodulatory potential of the crop	Biostimulant (type)	Extraction method	Aspects of the extraction method	Accumulated secondary metabolite(s) - reported result (yield/change)
*Achillea millefolium* L. (Asteraceae) (yarrow) (Bayat et al., 2021)	Enhances macrophage and lymphocyte activity; modulates cytokine production	Humic and fulvic acids (Bayat et al., 2021)	Methanolic extraction (Bayat et al., 2021)	Efficient for polar secondary metabolites, offers excellent extraction yields and reproducibility, it also presents limitations such as co-extraction of impurities, potential degradation of heat- or light-sensitive metabolites, and toxicity concerns that require complete solvent removal before biological assays (Michalak et al., 2015)	Total phenols - 59% and 57% more than the control (Bayat et al., 2021)
*Capsicum annuum* L. (Solanaceae) (pepper) (Bonini et al., 2020)	Enhances macrophage and lymphocyte responses; modulates Th1/Th2 cytokine balance	Microbial-based biostimulants (*Rhizoglomus irregularis, Funneliformis mosseae* and *Trichoderma koningii*) (Bonini et al., 2020)	Ultra-Turrax-assisted acidified methanolic extraction (Bonini et al., 2020)	Uses high-shear homogenization with 80% methanol acidified with 0.1% formic acid to rupture cells and release metabolites. Presents minimal thermal degradation. Requires methanol removal before assays. Best suited for polar metabolites; less effective for non-polar metabolites (Santos et al., 2025)	Vitamin A (1.5×), α-carotene (8.5×), blumenol B (2–2.5×), saponins (1.5–10×), and phenolics (3–87×) higher in biostimulant-treated plants compared to control (Bonini et al., 2020)
*Ocimum basilicum* L. (Lamiaceae*) (*sweet basil) (Deveikytė et al., 2025)	Enhances macrophage activity, lymphocyte proliferation, and antibody production in experimental models	Amino acids (isoleucine, methionine, glutamine, tryptophan and pheny-lalanine) (Deveikytė et al., 2025)	Ethanolic extraction (Deveikytė et al., 2025)	Effective for both polar and moderately non-polar secondary metabolites. Preserves thermolabile metabolites and aligns with green extraction principles. Acidified ethanol enhances phenolic and flavonoid yields	Total phenols presented a 5.8% increase compared to the controls (Deveikytė et al., 2025)
*Diplotaxis tenuifolia* L. (Brassicaceae) (perennial wall-rocket) (Caruso et al., 2019)	Phenolics and glucosinolate derivatives linked to modulation of oxidative and inflammatory pathways	Legume-derived protein hydrolysate (Caruso et al., 2019)	Methanomic extraction (Caruso et al., 2019)	Broad polarity range, easy to remove post extraction, high efficiency and yields	Presented a 11% increase in total phenols compared to the controls (Caruso et al., 2019)

Overall synthesis: major trend = biostimulant application consistently enhanced the accumulation of phenolics, carotenoids, glucosinolates, and other immunomodulatory secondary metabolites across diverse crop systems; dominant extraction pattern = methanolic and ethanolic extraction methods remained the most commonly applied approaches due to their high efficiency in recovering polar and moderately polar metabolites; key methodological limitation = although several extraction methods provided high metabolite yields and reproducibility, factors such as solvent toxicity, metabolite instability, thermal degradation, and limited recovery of non-polar metabolites may affect downstream biological applicability; translational constraint = increased metabolite yield does not inherently ensure improved immunomodulatory efficacy, as extraction efficiency must also preserve metabolite stability, bioavailability, and biological functionality; conceptual advancement = unlike conventional extraction-focused assessments, this table integrates extraction efficiency with metabolite preservation and downstream immunomodulatory relevance, providing a more translational framework for evaluating metabolite enhancement strategies.

From a comparative perspective, ultrasound-assisted extraction and microwave-assisted extraction improve mass transfer and cell wall disruption, resulting in higher yields within shorter timeframes. Whereas supercritical fluid extraction particularly using supercritical CO_2_ provides high selectivity and solvent-free extracts but requires high capital investment and technical expertise. Enzyme-assisted extraction enhances yield through targeted degradation of plant cell walls, although enzyme cost and specificity may limit scalability (Bitwell et al., 2023; Ghenabzia et al., 2023). Thus, while advanced techniques demonstrate clear advantages in efficiency and metabolite stability, their scalability and economic feasibility remain key challenges for large-scale or industrial application.

Regardless of the method employed, extraction efficiency depends on optimising parameters such as solvent polarity, temperature, extraction time, pressure, and plant pre-treatment, all of which influence metabolite recovery, stability, and reproducibility. Importantly, from both research and industrial perspectives, the selection of extraction technique must balance efficiency, metabolite stability, scalability, cost, and environmental sustainability. Consequently, improving extraction methods is essential not only for maximising metabolite yield but also for preserving bioactivity, ensuring reproducibility, and facilitating the translation of plant-derived metabolites into functional or therapeutic applications.

Table 3 also highlights some common immunomodulatory potentials of different crops that have been particularly treated for enhanced secondary metabolites. This is an important aspect as traditional medicinal plants play an important role in reducing communicable disease burden in developing regions, largely due to their immunomodulatory secondary metabolites (Wang et al., 2007). Common edible crops already consumed daily contain immune-active metabolites. For example, A*nnona muricata* L. (Annonaceae) (soursop) contains alkaloids, phenolics and megastigmanes, *Citrus* L. (Rutaceae) (citrus fruits) contain carotenoids, phenolic acids and vitamin C, whereas *Allium sativum* L. (Amaryllidaceae) (garlic) contains ascorbic and sulphur. Further, *Allium cepa* L. (Amaryllidaceae) (onions) contain flavonoids and organosulfurs, while *Daucus carota* (Apiaceae) carrots contain carotenoids (Cardelle-Cobas et al., 2010; Matsushige et al., 2012; Raiola et al., 2014; Yang et al., 2015). These widely consumed crops therefore represent important dietary sources of immunologically active phytochemicals, which exert their effects through specific molecular and cellular immune regulatory pathways.

These metabolites have been shown to influence immune function by modulating macrophage polarisation through major signalling axes, including IFN-γ-STAT1, TLR4-IRF5/NF-κB/AP-1, IL-4-STAT6/IRF4, PPARγ, and CREB/C/EBP (Sharma et al., 2017; Alikiaii et al., 2021; Sharma et al., 2024). Recent mechanistic studies further support the importance of immune-cell reprogramming in tissue repair and regenerative responses. For example, Zhou Z. et al. (2023) demonstrated that activation of the AMPK/PGC-1α/PPAR-γ signalling axis promoted macrophage polarisation toward the anti-inflammatory M2 phenotype, resulting in enhanced axonal regeneration, remyelination, and peripheral nerve recovery. The study highlighted that modulation of macrophage functional states plays a critical role in controlling inflammatory resolution and tissue regeneration. These findings are particularly relevant to medicinal plant-derived secondary metabolites, many of which have been reported to influence macrophage activation, cytokine production, and oxidative stress pathways. Consequently, enhancement strategies that increase accumulation of bioactive metabolites capable of regulating macrophage polarisation may contribute significantly to improved immunomodulatory and regenerative therapeutic outcomes.

Despite the recognised benefits of edible crops consumed daily, a critical gap remains in integrating biostimulant-induced metabolite enhancement with optimised extraction strategies, as inefficient extraction may limit the recovery and downstream utilisation of these metabolites. Bridging this gap is essential for maximising both agronomic and therapeutic value. Integrating biostimulant application with optimised extraction and processing strategies therefore represents a key step toward enhancing the availability, consistency, and functional efficacy of immune-active plant metabolites for both nutraceutical and pharmaceutical applications.

### 3.6 Synthesis of Section 3.1–3.4

Collectively, the evidence presented across Sections 3.1–3.4 demonstrates that biostimulant- induced enhancement of immunomodulatory potential in medicinal plants is governed by a highly coordinated interaction between signalling pathways, metabolic regulation, and physicochemical constraints. A consistent pattern emerges in which the phenylpropanoid, flavonoid, and terpenoid pathways are preferentially activated across diverse biostimulant types, with seaweed-based extracts particularly *Ascophyllum nodosum* showing the most reproducible effects across plant systems. However, this response is not universal, as metabolite accumulation is strongly modulated by genotype, environmental conditions, and application parameters, highlighting the context-dependent nature of biostimulant efficacy.

Importantly, pathway activation alone does not guarantee enhanced functional outcomes, as metabolic flux limitations, pathway bottlenecks, and trade-offs with primary metabolism can constrain yield. Furthermore, increased *in planta* accumulation does not necessarily translate into improved immunomodulatory efficacy, due to critical limitations associated with metabolites stability, bioavailability, and extraction efficiency, thereby revealing a key disconnect between biochemical enhancement and functional application. Therefore, a systems-level approach integrating pathway induction, metabolic flux optimisation, metabolite stabilisation, and advanced extraction strategies is required to achieve consistent and biologically relevant improvements in immunomodulatory activity. These insights highlight both the potential and the current limitations of biostimulant-based strategies and underscore the need for more mechanistically driven, standardised, and translational research frameworks. This integrative perspective advances current reviews by linking plant metabolic reprogramming with downstream pharmacological relevance, thereby providing a more holistic framework for developing biostimulant-driven medicinal plant systems.

### 3.7 Elicitors

Elicitors are molecules that activate defence-related hypersensitivity responses in plants, whereas biostimulants mainly promote general growth and development (Yoshikawa et al., 1993). Elicitors are considered highly effective triggers of secondary-metabolite biosynthesis because they manipulate key metabolic and biosynthetic pathways involved in specialised metabolism (Angelova et al., 2006; Thakur et al., 2019). Elicitors can be broadly classified as biotic or abiotic. Biotic elicitors include microorganisms (fungi and bacteria) and polysaccharides derived from yeast and mycelial cell walls such as chitin, pectin and cellulose (Ali Reza et al., 2021). Abiotic elicitors include physical factors (light, salinity, drought, osmotic and thermal stress), chemical factors (heavy metals) and plant hormones such as jasmonic acid, salicylic acid and gibberellic acid, typically applied at low concentrations (Yoshikawa et al., 1993).

The effectiveness of elicitors is highly context-dependent and varies significantly across plant species, developmental stages, and environmental conditions (Longkumer et al., 2025). This variability often leads to inconsistent outcomes in secondary metabolite accumulation, thereby limiting the predictability and scalability of elicitor-based strategies. For example, while jasmonic acid may strongly induce phenolic and alkaloid biosynthesis in some medicinal plants (e.g., increased total phenolics by 30%–60% and alkaloids by 20%–50% in *Catharanthus roseus* L. (Apocynaceae) (Martínez-Chávez et al., 2024). Similar treatments may produce negligible or even inhibitory effects in other species or under different environmental conditions such as drought or nutrient limitation (e.g., ≤10% change or reduced flavonoid content in *Ocimum basilicum* L. (Lamiaceae) (sweet basil) under water stress (Akula and Ravishankar, 2011; Martínez-Chávez et al., 2024). These inconsistencies highlight the need for species-specific optimisation and a deeper mechanistic understanding of elicitor-plant interactions.

Timing of elicitor application is crucial, as treatments applied during early growth phases may suppress biomass accumulation, whereas application during the late exponential phase often optimises both growth and secondary metabolite production (Guru et al., 2022). In addition, elicitor type and dosage play a decisive role due to their dose-dependent effects. While low concentrations may effectively stimulate secondary metabolism, higher concentrations can induce phytotoxicity, resulting in reduced growth or even cell death (Guru et al., 2022). Environmental and culture conditions including light intensity, temperature, and nutrient availability also significantly influence elicitor efficacy. For instance, exposure to UV-B radiation (290–320 nm) can enhance phenolic metabolite accumulation. However, its effectiveness is highly dependent on exposure duration and intensity (Thakur et al., 2019). Collectively, these factors highlight the complexity of elicitor-plant interactions and reinforce the need for carefully optimised, system-specific application strategies.

Importantly, elicitor-induced responses can be broadly categorised into transient induction effects and long-term metabolic reprogramming. Transient responses are characterised by rapid but short-lived increases in secondary metabolite production. This often linked to immediate defence signalling and activation of key enzymes such as phenylalanine ammonia-lyase (Guru et al., 2022). In contrast, long-term metabolic reprogramming involves sustained alterations in gene expression, enzyme activity, and metabolic fluxes, which may persist throughout plant development or even across generations. Such long-term effects are frequently associated with epigenetic modifications, including DNA methylation and histone modifications, which stabilise induced metabolic states (Devika et al., 2021; MacDonald and Mohan, 2025). However, the distinction between these two response types is often overlooked, leading to overestimation of elicitor efficacy in practical applications. While many studies report significant increases in metabolite accumulation shortly after elicitor application, these effects are not always maintained over time or under field conditions. Therefore, future research should prioritise understanding the durability of elicitor-induced responses and identifying conditions under which transient induction can be converted into stable metabolic reprogramming.

#### 3.7.1 *In vivo* elicitor applications

Application methods of elicitors differ, for examples in the *in vivo* applications, foliar spraying of either jasmonic acid, salicylic acid, chitosan, yeast extract is applied directly to the leaves. Thus, enhancing plant resistance to diseases and improving nutrient uptake (Awang et al., 2013). Other *in vivo* elicitor applications include (1) soil drenching which targets root-associated signalling and root absorption. This enhances their uptake and initiating defence responses in the root system and subsequently throughout the plant (Chamkhi et al., 2021). (2) Seed treatment or seed priming which enhances seed germination, early vigour and primes the plant for stronger defence responses (Pereira et al., 2024). (3) Trunk or stem injection which allows for precise delivery of the elicitor to the plant’s vascular system, ensuring efficient transport and uptake (Archer et al., 2022). (4) Hydroponic or nutrient solution enrichment, this allows for uniform distribution and absorption, suitable for controlled environment. This controlled nutrient solution’s composition offers greater control over plant growth, potentially leading to accumulation of secondary metabolites compared to traditional soil-based farming (Mehdizadeh and Moghaddam, 2024). (5) Gaseous or volatile application which are non-invasive, affects whole plant systemically (Xiao et al., 2022).

##### 3.7.1.1 Seed priming as an *in vivo* elicitor strategy for secondary metabolite enhancement

Seed priming represents a promising, yet comparatively underexplored *in vivo* strategy relative to foliar sprays and drench-based applications. Priming rapidly activates multi-level biological adjustments including enzymatic (Renu, 2018), hormonal (Rhaman et al., 2021), physiological, transcriptional and metabolomic reprogramming (Jatana et al., 2024; MacDonald and Mohan, 2025). Seed priming can be achieved through phytochemical (chemical) treatments, where seeds are soaked in aqueous solutions containing bioactive compounds such as potassium nitrate, calcium chloride, selenium, or zinc oxide nanoparticles before sowing (Mahmoudi et al., 2019; Abbasi-Khalaki et al., 2021; Abou-Zeid et al., 2021; Adhikary et al., 2022). These compounds act as biochemical cues that pre-activate defence signalling and key metabolic pathways, which can subsequently channel more carbon and precursor pools toward secondary metabolite biosynthesis during early plant development.

Chemical priming can also induce heritable epigenetic changes, including shifts in DNA methylation and histone acetylation, which may result in long-term improvements in plant performance across generations (Devika et al., 2021; MacDonald and Mohan, 2025). For instance, treating *Camptotheca acuminata Decne* (Nyssaceae) (happy tree) seeds with 60 mg/L choline chloride significantly enhanced the accumulation of the alkaloids camptothecin and 10-hydroxycamptothecin in young leaves (Zeng et al., 2012). These alkaloids are potent topoisomerase I inhibitors with established anticancer activity, particularly against colon, ovarian, and small-cell lung cancers (Hu et al., 2011; Fadaeinasab et al., 2020; Liu et al., 2024). Importantly, the therapeutic significance of these enhanced metabolites extends beyond direct pharmacological activity, as increasing evidence suggests that plant-derived bioactive metabolites can also modulate immune function through systemic and microbiota-associated mechanisms.

In this context, recent evidence further suggests that the immunomodulatory activity of plant secondary metabolites is closely associated with their ability to regulate the gut microbiota and intestinal immune environment. Su et al. (2023) reported that bioactive metabolites derived from *Astragalus* L. (Fabaceae) (locoweed), including polysaccharides, saponins, and flavonoids, improved intestinal barrier integrity, reduced chronic inflammation, and enhanced immune regulation through modulation of gut microbial composition and metabolism. The authors highlighted that alterations in intestinal microbiota influence the production of short-chain fatty acids, bile acid metabolism, endotoxin release, and inflammatory signalling pathways associated with immune dysfunction and metabolic disorders. Furthermore, *Astragalus* L. (Fabaceae) (locoweed)-derived metabolites were shown to reduce insulin resistance, suppress inflammatory responses, and improve immune homeostasis through coordinated effects on intestinal permeability, cytokine regulation, and microbial balance (Liang et al., 2024). These findings provide important mechanistic evidence supporting the concept that enhanced accumulation of medicinal plant secondary metabolites may improve immunomodulatory efficacy not only through direct immune-cell interactions, but also indirectly through microbiota-mediated regulation of host immunity and systemic inflammation.

Similarly, hormonal seed priming is gaining recognition as an effective approach for improving stress tolerance and secondary metabolite production. Plant hormones (phytohormones) act as key chemical messengers that regulate development and stress responses (Matilla-Vazquez and Matilla, 2014). Commonly used phytohormones for priming include auxins, cytokinins, gibberellins, abscisic acid, salicylic acid, ethylene, and jasmonates (Sytar et al., 2018; Ali Reza et al., 2021). Their effects vary with species, timing, and concentration, but they broadly influence stress signalling, cell division, photosynthesis, and metabolic regulation (Vob et al., 2014; Kazan, 2015). For example, exogenous cytokinin application in *Epipremnum aureum* (Araceae) increased carbon assimilation, net photosynthesis, and biomass (Di Benedetto et al., 2015). Further, medicinal crops such as *Nicotiana tabacum* L. (Solanaceae) (tobacco) and *Solanum melongena* L. (Solanaceae) (eggplant) have also shown improved responses to cytokinin and gibberellin priming, including increases in bioactive metabolites like solanesol (Edeke et al., 2021; Prommaban et al., 2022; Zhang et al., 2024). Collectively, these findings highlight the potential of hormonal seed priming as a strategic tool to enhance both stress resilience and secondary metabolite biosynthesis, although its effectiveness remains highly dependent on species-specific responses and optimization of application conditions.

#### 3.7.2 *In vitro* elicitor applications for secondary metabolite enhancement via plant cell culture techniques

Plant cell and tissue culture techniques offer environmentally sustainable alternatives to produce secondary metabolites, particularly when natural sources are scarce or chemical synthesis is impractical. These methods are especially advantageous because certain bioactive metabolites are only expressed or accumulated in specific tissues or under particular environmental conditions. Additionally, some plant species are either difficult to cultivate or require several years to mature. In contrast, cell culture enables the rapid, large-scale propagation of true-to-type plants in a short period, with minimal impact on natural ecosystems (Verpoorte et al., 2002). In this context, cell culture systems are highly beneficial, as they support year-round plant production under aseptic and controlled conditions (Ozyigit et al., 2023). This continuous cultivation eliminates seasonal limitations, allowing consistent and scalable production of secondary metabolites (Bulgakov et al., 2010).

Moreover, isolating secondary metabolites from cultured cells is often more efficient, reliable, and faster than extraction from wild populations (Verpoorte et al., 2002; Kolewe et al., 2008). Cell cultures also provide a platform for integrating advanced biotechnological approaches, such as metabolic engineering and conventional elicitor applications, to further enhance metabolite yields (Bulgakov et al., 2010; Khani et al., 2012). However, despite these advantages, several critical limitations constrain the widespread application of *in vitro* culture systems.

High production costs associated with sterile conditions, specialised infrastructure, skilled labour, and energy-intensive bioreactor operations remain a major barrier to commercial scalability. Furthermore, while cell suspension cultures are often described as scalable, translating laboratory-scale success to industrial-scale production is complex and frequently limited by issues such as shear sensitivity, oxygen transfer inefficiencies, and inconsistent metabolite yields in large bioreactors (Verpoorte et al., 2002; Kolewe et al., 2008). Genetic and metabolic instability during prolonged subculturing also presents a significant challenge, as somaclonal variation may lead to fluctuations or declines in secondary metabolite production over time. In addition, regulatory constraints associated with the use of genetically modified cell lines, particularly in pharmaceutical and nutraceutical applications, can further limit commercial adoption. These challenges highlight the need for improved bioprocess optimisation, stable culture systems, and clear regulatory frameworks to fully realise the potential of plant cell culture technologies.

The process of cell culture begins with biomass generation, involving the selection of suitable explants (e.g., leaves, stems, roots), surface sterilization, and callus induction on nutrient media. The media is supplemented with plant growth regulators such as auxins and cytokinins (Ozyigit et al., 2023). Calli are subsequently transferred to liquid media to establish suspension cultures, enabling scalable and homogeneous biomass production. At this stage the suspension cultures are particularly prone to variability in growth kinetics and metabolite accumulation, especially under suboptimal culture conditions, further complicating scale-up and process standardisation. As an alternative, hairy root cultures, induced by *Agrobacterium rhizogenes*, offer a highly efficient alternative, producing fast-growing, genetically stable roots that often accumulate secondary metabolites at levels comparable to or exceeding intact plants.

These *in vitro* systems maintain high metabolic activity, phenotypic consistency, and hormone-independent growth, making them suitable for long-term production of alkaloids, phenolics, and terpenoids, as well as for integrating elicitation or metabolic engineering strategies (Srivastava and Srivastava, 2012; Mehrotra et al., 2018). Nevertheless, even hairy root systems face limitations related to large-scale bioreactor design, oxygen diffusion, and regulatory acceptance, particularly when derived via genetic transformation.

As the above cultures reach the stationary growth phase, stress-inducing conditions such as reduced nitrogen or phosphate availability are induced thus triggering secondary metabolite synthesis (Berlin et al., 1989). Production can be further enhanced through precursor feeding, elicitation, biotransformation, cell immobilization, and permeabilization, which optimize metabolic flux, stimulate defence-related pathways, and facilitate metabolite recovery (Brodelius and Nilsson, 1983; Bhatia et al., 2015; Busto et al., 2008). These approaches have markedly increased yields of key metabolites, including solanidine, solasodine, and ajmalicine, while maintaining cell viability (Brodelius and Nilsson, 1983; Lee et al., 2007). By providing a controlled environment that overcomes the low natural abundance of bioactive metabolites, plant cell culture systems represent a promising platform for producing secondary metabolites with potent immunomodulatory properties. Therefore, plant cell culture systems represent a promising platform for producing secondary metabolites with potent immunomodulatory properties. However, the long-term sustainability and commercial viability of these systems depend on overcoming existing technical, economic, and regulatory challenges. Importantly, the biological relevance of these enhanced metabolites may extends beyond production efficiency, as their functional roles in modulating immune responses and inflammatory environments are increasingly being recognised.

Recent advances in cancer immunology further reinforce the importance of immune microenvironment regulation as a key determinant of therapeutic efficacy and disease progression. Importantly, Wang et al. (2024) highlighted that the tumour immune microenvironment plays a central role in shaping immune responsiveness particularly in immunologically “cold” tumours such as ovarian cancer, where immune cell dysfunction and metabolic reprogramming contribute to poor immunotherapy outcomes. The authors further emphasised that reprogramming of immune cell populations and their metabolic states is critical for restoring immune activity within suppressive microenvironments. Similarly, Xu et al. (2024) reported that metastatic renal cell carcinoma patients with elevated FHL2 expression exhibited enhanced immune infiltration but also showed increased CD8^+^ T-cell exhaustion and immunosuppressive tumour microenvironment characteristics, resulting in poorer responsiveness to combined immunotherapy and tyrosine kinase inhibition. The study emphasised that immune-cell functional status, rather than immune-cell abundance alone, is a critical determinant of therapeutic efficacy. These concepts are directly relevant to plant-derived secondary metabolites, many of which have been shown to modulate immune cell activation, macrophage polarisation, and inflammatory signalling pathways. Consequently, enhancement of bioactive metabolite accumulation in medicinal plants may contribute not only to systemic immunomodulation but also to the reprogramming of dysfunctional immune microenvironments, thereby improving therapeutic responsiveness.

### 3.8 Synthesis of Section 3.5

Collectively, the evidence across elicitor-based strategies highlights their role as highly targeted regulators of secondary metabolism, capable of inducing both rapid defence responses and long-term metabolic reprogramming. A key pattern is the consistent involvement of signalling molecules such as jasmonic acid and salicylic acid in activating phenylpropanoid-, flavonoid-, and alkaloid-associated pathways. Although the magnitude and stability of these responses remain highly species- and context-dependent. In contrast to biostimulants, elicitors exhibit greater specificity but also greater variability, with outcomes strongly influenced by dosage, timing, developmental stage, and environmental conditions, thereby limiting predictability and scalability. Comparative evaluation across elicitor systems further indicates that reproducibility remains a major unresolved limitation in the field, as many reported responses are highly conditional and insufficiently validated across environments, developmental stages, and species.

Importantly, the distinction between transient induction and stable, epigenetically mediated metabolic reprogramming represents a critical but often overlooked factor determining long-term efficacy. Across application systems, *in vivo* approaches such as foliar application and seed priming offer practical and low-cost strategies. With seed priming emerging as a particularly promising tool due to its capacity to induce early-stage metabolic programming and potential heritable effects. Conversely, *in vitro* systems provide highly controlled environments for maximising metabolite production and enabling biotechnological interventions, but are constrained by scalability, cost, and regulatory limitations. This contrast underscores a fundamental trade-off between control and applicability across elicitor-based systems. Therefore, future progress in elicitor-driven enhancement of immunomodulatory metabolites will depend on integrating precise signalling manipulation with optimised delivery strategies and scalable production platforms. This integrative perspective advances current approaches by distinguishing between short-term induction and stable metabolic reprogramming. While linking elicitor application strategies with both agronomic feasibility and downstream pharmacological relevance.

## 4 Genetic strategies to enhance secondary metabolite accumulation and immunomodulatory potential in medicinal plants

### 4.1 Convectional breeding

Sleper and Poehlman (2006) defined plant breeding as “the art and science of improving the heredity of plants for the benefit of humankind.” More broadly, plant breeding can be described as the targeted manipulation of plant genetic patterns, genetic makeup, or genome to produce offspring with desirable traits (Moose and Mumm, 2008). Thereby increasing their value and contributing to human wellbeing. Traditional plant breeding methods such as hybridization and selection have long been employed to enhance various traits, including the production of secondary metabolites. One approach involves selecting naturally high-performing genotypes that are rich in the desired secondary metabolites (Fehr, 1987; Mashilo et al., 2018; Mkhize et al., 2023a). Breeders also typically cross these high-performing individuals to develop progeny with superior traits (Fehr, 1987). The success of such crosses depends on the availability of suitable genetic variation and effective selection strategies.

While conventional breeding remains a foundational strategy, its practical implementation in medicinal plants is constrained by several factors. The polygenic nature of secondary metabolite biosynthesis complicates trait selection, as these pathways are often regulated by multiple genes and are highly influenced by environmental conditions. As a result, phenotypic selection may not consistently translate into stable metabolite expression across environments, raising concerns regarding trait stability and reproducibility (van de Wiel et al., 2010). In some instances, heterosis (hybrid vigor) is observed, where the offspring exhibit significantly higher performance compared to their parents. Another challenge is the need for genotypes displaying desirable traits to be further assessed for stability across diverse environments, which supports the selection of candidates for cultivar release (Acquaah, 2015). However, achieving stable expression of secondary metabolites remains challenging, as genotype × environment interactions can significantly alter phytochemical profiles. This variability limits the reliability of conventionally bred cultivars for consistent pharmaceutical or nutraceutical applications, where standardisation is critical.

Although conventional breeding methods are effective, they are time-consuming and rely heavily on the existing natural variation within the germplasm. From a feasibility perspective, long breeding cycles and the need for multi-location trials increase both time and financial investment, making conventional breeding less attractive for rapid metabolite enhancement compared to biotechnological approaches. Additionally, the use of wild genotypes with limited genetic information in breeding programmes, as is often the case with medicinal plants, presents both challenges and opportunities. The lack of genomic data makes trait inheritance unpredictable, complicates selection due to environmental influence on phenotypes, and increases the risk of linkage drag, where undesirable traits are co-inherited with beneficial ones. Also, ethical and conservation considerations arise when exploiting wild germplasm, particularly in biodiversity-rich regions, where overharvesting or bioprospecting without equitable benefit-sharing may raise regulatory concerns. In contrast, wild genotypes offer rich genetic diversity and often possess unique alleles for stress tolerance, disease resistance, and enhanced secondary metabolite production (Mkhize et al., 2023b). These traits can be invaluable for improving the medicinal and immunomodulatory properties of cultivated plants. Despite the complexities, incorporating wild genotypes remains a powerful strategy to broaden the genetic base and introduce novel traits into breeding programs.

Several studies mentioned in Table 4 have employed traditional breeding approaches to enhance the accumulation of secondary metabolites, many of which are known for their immunomodulatory properties. The metabolites mentioned in Table 4 can influence key immune processes such as the proliferation and differentiation of immune cells, as well as the secretion of cytokines and chemokines, molecules essential for coordinating immune function. Additionally, they may modulate the activity of natural killer cells, which play a vital role in combating infections (Wang et al., 2013; Ge et al., 2022). Phenolic acids, in particular, have been shown to interact with various signalling pathways involved in inflammation and immune regulation (Miranda-Rottmann et al., 2002). Therefore, by identifying and combining high-performing genotypes rich in bioactive metabolites, breeders can develop cultivars with improved immunomodulatory properties that support key immune functions.

**TABLE 4:** Examples of traditional breeding targeting increased accumulation of secondary metabolites.

Breeding approach	Targeted secondary metabolites	Crop	References
Selection and hybridization for high pigment alleles	Lycopene, β-carotene and flavonoids	*Solanum lycopersicum* L. (tomato)	George et al. (2004); Kolotilin et al. (2007); Gonzali and Perata, (2020)
Mass selection and recurrent selection	Carotenoids (β-carotene, lutein)	*Daucus carota* L. (Apiaceae) (carrot)	Riaz et al. (2022)
Selection from landraces with deep pigmentation	Anthocyanins	*Zea mays* L*.* (Poaceae) (purple corn)	Miranda-Rottmann et al. (2002)
Breeding for pigmented rice varieties (black, red)	Phenolics, flavonoids and γ-oryzanol	*Oryza sativa* L. (Poaceae) (rice)	Walter and Marchesan (2011)
Selection and crossing of coloured grain types	Phenolic acids, alkylresorcinols and anthocyanins	*Triticum aestivum* L. (Poaceae) (wheat)	Ficco et al. (2014)
Pedigree breeding, backcrossing	Capsaicinoids, carotenoids and flavonoids	(*Capsicum annuum L.* (Solanaceae) (chili pepper)	Padilha and Barbieri, (2016); Lozada et al. (2022)
Hybridization and recurrent selection	Glucosinolates and phenolic metabolites	*Brassica oleracea* (Brassicaceae) (broccoli)	Han et al. (2021); Roberto Lo Scalzo et al. (2024)
Selection and hybridization	Isoflavones (e.g., genistein, daidzein)	*Glycine max* (L.) Merr. (Fabaceae) (soybean))	Kim et al. (2014); Miladinović et al. (2019)
Clonal selection and hybrid development	Catechins, theaflavins and polyphenols	*Camellia sinensis* L. Kuntze (Theaceae) (tea)	Wei et al. (2011); Leonida et al. (2013)
Recurrent selection and crossing	Essential oils (e.g., linalool and eugenol)	*Ocimum basilicum* L. (Lamiaceae) (basil))	Šovljanski et al. (2022)

### 4.2 Marker-assisted selection

Marker-Assisted Selection (MAS) expedites the breeding process by utilizing molecular markers linked to genes involved in the biosynthesis of secondary metabolites (Kumar et al., 2024). Marker-Assisted Selection enhances selection accuracy, shortens breeding cycles, and is therefore a valuable tool for trait improvement in medicinal plants. For example, markers associated with artemisinin production in *Artemisia annua* L. (Asteraceae) (sunflower) and alkaloid biosynthesis in *Catharanthus roseus* (L.) G.Don (Apocynaceae) (periwinkle) have facilitated the efficient identification and selection of high-yielding lines (Barut et al., 2022). These examples highlight the effectiveness of Marker-Assisted Selection in accelerating and refining the breeding of medicinal plants.

The process of MAS typically begins with the selection of parental lines exhibiting contrasting levels of target metabolites. This is followed by the development of a mapping population. Metabolite concentrations are then quantified using advanced analytical techniques such as HPLC or GC-MS, while genotyping is performed using molecular markers like SSRs and SNPs (Theodoridis et al., 2008; Theodoridis et al., 2012; Bell et al., 2016; Wishart, 2016). Linkage maps are constructed to identify markers associated with traits of interest, and QTL mapping is employed to locate genomic regions regulating metabolite biosynthesis. Validated markers are subsequently used to screen breeding populations for desirable alleles. Thereby accelerating the development of elite genotypes with enhanced metabolite profiles. The resulting data from metabolite profiling and genotyping are further analysed using statistical tools such as Principal Component Analysis (PCA) and Batch-Learning Self-Organizing Mapping (BL-SOM). These techniques display data as a lattice of coloured cells, distinguishing plant samples based on variations in metabolite content. Notably, BL-SOM is also used to detect chemical marker peaks, with each cell reflecting increases or decreases in peak intensities, enabling high-resolution differentiation (Okada et al., 2009). This integrative approach offers a precise, rapid, and cost-effective method for trait selection, minimizes environmental variability, and allows for early-stage screening.

Metabolite profiling and functional assessments such as antioxidant and anti-inflammatory activity have been conducted on various medicinal plants using platforms like HPLC, GC-MS, and NMR. These include *Aegle marmelos* L. (Rutaceae) (citrus) (Tiwari et al., 2021), *Terminalia catappa* L. (Combretaceae) (almond) fruit peels (Kaneria et al., 2018), *Swertia chirayita* (Roxb.) H. Karst. (Gentianaceae) (chirata), *Swertia mussotii Franch* (Gentianaceae) (tibetan) (Fan et al., 2014), and *Ephedra pachyclada Boiss* (Ephedraceae) (mormon-tea) (Okada et al., 2009). In *Catharanthus roseus* (L.) G.Don. (Apocynaceae) (periwinkle), MAS has facilitated the improvement of alkaloid production, particularly of pharmacologically important metabolites such as vinblastine and vincristine, known for their anticancer properties (Rai et al., 2022). Furthermore, Random Amplified Polymorphic DNA markers have been used to distinguish species such as *Allium schoenoprasum* L. (Amaryllidaceae) (chives), *Codonopsis pilosula* L. (Campanulaceae) (bellflower), and *Andrographis paniculata* L. (Acanthaceae) (bitter weed) (Hasan et al., 2024). Inter-Simple Sequence Repeat markers have also been employed to differentiate *Arabidopsis thaliana* L. (Brassicaceae) (thale cress) genotypes (Barth et al., 2002). In *Withania somnifera (L.) Dunal.* (Solanaceae) (ashwagandha), MAS has been applied to enhance the concentration of withanolides, a bioactive metabolites with wide-ranging therapeutic applications (Arya et al., 2025; Chawla et al., 2025). These studies contribute to the identification and quantification of genetic diversity within medicinal plant populations, offering a strategic framework for selecting and breeding genotypes with optimal secondary metabolite content and diversity, particularly those with potential for industrial and pharmaceutical utilization.

Despite its advantages, MAS has limitations in addressing complex traits such as secondary metabolite accumulation, which are often controlled by multiple quantitative trait loci (QTLs) and influenced by environmental interactions (Kumar et al., 2024). This reduces the predictive power of markers and may limit their effectiveness across diverse genetic backgrounds. The success of MAS depends heavily on the availability of well-characterised genomes, high-density marker systems, and robust phenotyping platforms, which are often lacking for many medicinal plant species. While Marker-Assisted Selection improves the efficiency of conventional breeding, it remains limited in its ability to manipulate complex metabolic pathways directly. Consequently, more advanced genetic approaches, including genome editing and transgenic technologies, are increasingly being explored to enable precise modification of biosynthetic pathways. These approaches offer greater specificity and control over metabolite production but are also associated with technical, regulatory, and ethical challenges, which are discussed in the following section.

### 4.3 Transgenic approaches

Transgenic approaches have been effectively employed as breeding strategies to enhance the accumulation of bioactive metabolites in plants. These include genetic transformation, gene overexpression, and RNA interference (RNAi)-based gene silencing. When a target gene or trait is absent in parental lines or related species, transformation technologies can be used to introduce genes from other organisms into new plant varieties (Saito et al., 1992; Sharma and Maheshwari, 2015). Among the most widely used methods are *Agrobacterium*-mediated transformation and biolistic (gene gun) techniques, both of which facilitate the delivery of genes encoding critical enzymes or regulatory proteins (Saito et al., 1992; Wang and Kin-Ying, 2004; Niazian et al., 2019). *Agrobacterium* plasmids serve as natural gene vectors containing circular DNA, which can be modified through restriction digestion to insert a gene of interest, commonly referred to as T-DNA into the plant genome (Saito, 1994). Both tissue culture-based and *in planta Agrobacterium*-mediated transformation techniques have been applied to improve stress tolerance and metabolite accumulation in medicinal plants. For instance, the introduction of the betaine aldehyde dehydrogenase (*BADH*) gene in *Trachyspermum ammi* (L.) Sprague, Apiaceae (ajowan) enhanced tolerance to drought and salinity and led to increased accumulation of bioactive metabolites such as *p*-cymene and thymol (Niazian et al., 2019). Similarly, Wang and Kin-Ying (2004) reported the first successful *Agrobacterium*-mediated transformation in *Echinacea purpurea* (L.) Moench (asteraceae), a high-value medicinal plant, which resulted in elevated levels of multiple bioactive metabolites.

Transgenic plants engineered to overexpress transcription factors such as *ORCA*3 or biosynthetic enzymes like squalene synthase have demonstrated significant increases in the accumulation of target secondary metabolites (Lee et al., 2004). Similarly, the industrial *Papaver somniferum* L. (Papaveraceae) (opium poppy) has been genetically modified to enhance morphinan alkaloid production through the introduction of the berberine bridge enzyme gene (Frick et al., 2005). Such targeted overexpression strategies boost metabolic flux through specific biosynthetic pathways, while RNAi-mediated gene silencing can suppress competing or inhibitory pathways, thereby further promoting the accumulation of desired bioactive metabolites (Bharti et al., 2023; Al Abound, 2024). A notable example is the jasmonate- and salicin-responsive *WRKY* transcription factor (*WsWRKY1*) in *Withania somnifera* (L.) Dunal (Solanaceae) (Ashwagandha) silencing *WsWRKY1* led to impaired plant growth and significantly downregulated genes in the phytosterol biosynthetic pathway, resulting in decreased levels of both phytosterols and withanolides. Conversely, overexpression of *WsWRKY1* enhanced the accumulation of these important triterpenoids (Singh et al., 2017). In another example, *Papaver somniferum* L. (Papaveraceae) (breadseed poppy) was subjected to RNA interference to silence the codeinone reductase (*COR*) gene family using a chimeric hairpin RNA construct. This approach led to the high-yield accumulation of reticuline, a non-narcotic alkaloid, by diverting the biosynthetic pathway away from narcotic end-products (Allen et al., 2004). Despite these promising advances, a significant challenge in medicinal plant breeding remains the limited availability of elite genetic resources. Many medicinal species are derived from wild populations or primitive cultivars. While wild populations offer a valuable reservoir of genetic traits for transgenic breeding, their use is limited by genomic unpredictability, ecological concerns, and technical barriers to transformation and gene expression stability.

### 4.4 Mutation breeding

The spontaneous mutation rate in plants can be significantly increased using radiation or chemical mutagens. Commonly used chemical mutagens include ethyl methane sulfonate (EMS) and sodium azide (Devi et al., 2023; Reshi et al., 2023). These agents induce changes in the plant genome, causing the resulting mutants to exhibit phenotypic differences compared to their non-mutated counterparts. Induced mutations generate novel genetic variability, either within the mutants themselves or in their progeny when crossed with other genotypes. Such mutagenic treatments can also modify gene expression, leading to alterations in metabolic pathways and potentially enhancing the biosynthesis of specific secondary metabolites (Nikalje et al., 2024).

Plants exhibiting advantageous mutant traits may be preferentially selected due to their enhanced fitness, conferred by these novel adaptive features. For example, a high-alkaloid variety of periwinkle *Catharanthus roseus* (L.) G. Don, (Apocynaceae) named “Dhawal” was developed through chemical mutagenesis and showed superior performance over the earlier “Nirmal” variety (Kolakar et al., 2018). Similarly, CRISPR/Cas9-mediated (Clustered Regularly Interspaced Short Palindromic Repeats and CRISPR-associated) mutagenesis of the *VvbZIP36* gene in*Vitis vinifera* L. (Vitaceae) (grapevine). led to increased anthocyanin accumulation, demonstrating the potential of targeted mutation in enhancing metabolite production (Tu et al., 2022). In another study, EMS-induced periwinkle mutants evaluated by Baskaran et al. (2013) showed elevated levels of the medicinal alkaloids vinblastine and vincristine, despite a reduction in overall yield. In *Trigonella foenum-graecum* L. (Fabaceae) (pea), treatment of seeds with gamma rays, EMS, and ethylene imine produced 11 improved lines with enhanced yields and higher diosgenin content. Additionally, mutation breeding using gamma irradiation and EMS has been applied to generate non-opium-producing varieties of the opium poppy *Papaver somniferum* L. (Papaveraceae (opium poppy) (Hasan et al., 2024). By inducing genetic variability through chemical or physical mutagens, researchers can uncover or amplify metabolic pathways responsible for the biosynthesis of bioactive metabolites. Such induced mutations may activate dormant genes or alter regulatory networks, leading to increased production of specific secondary metabolites. These mutant lines, once identified and stabilized, can be selectively bred or used as elite genotypes in developing high-value medicinal crops tailored for therapeutic applications.

### 4.5 Alternative breeding approaches to enhance secondary metabolites accumulation and immunomodulatory potential in medicinal plants

Other genetic approaches for enhancing secondary metabolite accumulation in medicinal plants include advanced technologies such as genome editing tools, most notably the CRISPR/Cas systems as well as integrative methods like omics and systems biology, synthetic biology, and metabolic engineering (Reshi et al., 2023; Jia et al., 2024). Among these, CRISPR/Cas systems have emerged as powerful tools that enable precise modification of endogenous genes involved in secondary metabolism (Feng et al., 2013; Gholizadeh et al., 2020). Through editing of promoter regions to upregulate gene expression or knocking out repressor genes, CRISPR/Cas can significantly influence metabolite production. This system utilizes a guide RNA to direct an enzyme, commonly Cas9, to a specific DNA sequence, where it introduces a cut (Liu and Fan, 2014). This targeted cut can then be harnessed to disable a gene, insert a new gene, or implement other specific genetic alterations (Liu and Fan, 2014; Kumar and Jain, 2015).

The CRISPR/Cas mechanism functions through three main stages: adaptation, expression, and interference (Makarova et al., 2011). During the adaptation stage, approximately 30 bp fragments of foreign DNA (called protospacers) are integrated into the CRISPR locus at the leader end, with selection guided by protospacer adjacent motifs (PAMs). In the expression stage, the CRISPR array is transcribed into a long precursor CRISPR RNA (pre-crRNA), which is then processed into individual mature crRNAs. Finally, during the interference stage, the crRNA forms a complex with Cas proteins to guide sequence-specific recognition and cleavage of the invading nucleic acid, typically resulting in a double-strand break (Mao et al., 2013).

A notable example of this application is seen in *Salvia miltiorrhiza* Bunge (Lamiaceae) (red sage), where CRISPR/Cas9 was used to target the *CYP76AH1* gene involved in tanshinone biosynthesis, leading to enhanced secondary metabolite accumulation (Contreras et al., 2019). Furthermore, Nora and Al Aboud (2024) compiled a comprehensive list of transcriptional regulators associated with secondary metabolite biosynthesis, clearly categorizing them as either activators or repressors. Their work sheds light on core regulatory elements that initiate or suppress specific metabolic pathways and illustrates how these factors shape metabolite profiles. This provides a strategic platform for genetic interventions aimed at boosting secondary metabolite production, especially those with immunomodulatory properties.

### 4.6 Synthesis of Section 4

Collectively, the genetic strategies discussed across Sections 4.1–4.5 reveal a continuum of approaches ranging from broad, phenotype-driven selection to highly precise genome-level interventions for enhancing secondary metabolite accumulation and immunomodulatory potential in medicinal plants. A key pattern is that conventional breeding provides a valuable foundation for exploiting natural genetic variation, yet is constrained by polygenic inheritance, genotype × environment interactions, and limited predictability of metabolite expression. Marker-assisted selection partially overcomes these limitations by improving selection efficiency and enabling early screening of desirable traits. However, its effectiveness remains restricted for complex metabolic traits governed by multiple quantitative loci and environmental influences. In contrast, transgenic and mutation-based approaches offer more direct manipulation of biosynthetic pathways, enabling targeted enhancement of key metabolites through gene overexpression, silencing, or induced variability. Although these methods introduce challenges related to genetic stability, regulatory approval, and public acceptance. Emerging genome editing technologies, particularly CRISPR/Cas systems, represent a significant advancement by enabling precise, predictable, and efficient modification of genes and regulatory elements controlling metabolic pathways.

Despite these advances, a critical limitation across all genetic strategies is the insufficient integration of metabolic regulation with downstream functional outcomes. As enhanced metabolite accumulation does not always translate into consistent immunomodulatory efficacy due to pathway complexity and post-synthetic constraints. This review therefore advances existing literature by critically integrating genetic engineering precision with downstream metabolic functionality and translational immunopharmacological constraints, rather than evaluating genetic strategies solely on enhancement efficiency. Therefore, future progress requires a systems-level integration of genetic, metabolic, and functional analyses, combining conventional and advanced breeding approaches with omics-driven insights and pathway engineering. This integrative perspective advances current frameworks by linking genetic manipulation of secondary metabolism with both biochemical optimisation and pharmacological relevance, thereby providing a more holistic strategy for developing medicinal plants with enhanced and consistent immunomodulatory properties.

## 5 Influence of agronomic practices, environmental conditions, and post-harvest treatments on enhanced secondary metabolite accumulation and immunomodulatory potential in medicinal plants

Practices that induce stress in plant growth can stimulate the synthesis of secondary metabolites. These include agronomic practices, environmental and controlled growth conditions, as well as post-harvest induction techniques. For instance, establishing the correct balance in fertilizer application is essential, as nutrient stress is known to enhance secondary metabolism (Feng et al., 2002). One study reported that a short-term treatment with an NH_4_^+^-N/NO_3_^−^-N ratio of 25:75 resulted in higher camptothecin content in young leaves by enhancing tryptophan decarboxylase activity in the stem bark of *Camptotheca acuminata Decne* (Nyssaceae) (happy tree) seedlings (Sun and Yan, 2008). Another effective strategy involves the use of micronutrients such as zinc and selenium, which act as cofactors in enzymatic pathways. For example, zinc is a cofactor for enzymes involved in the biosynthesis of tryptophan, an amino acid precursor to several important secondary metabolites (Gunes et al., 2011; Kaur et al., 2014). Selenium, a beneficial element, has been shown to enhance photosynthesis, antioxidant metabolism, carbohydrate accumulation, and the production of secondary metabolites in plant leaves (Andrade et al., 2018).

Crop density and light management also play crucial roles in promoting secondary metabolite accumulation. Adjusting planting density or using light filters to modify light intensity and spectrum can influence the production of phenolics and pigments. In one study, higher planting density led to an increased concentration of total cannabidiol and altered terpene distribution (Reichel et al., 2024). Notably, uniform light distribution across the plant canopy was a key factor, resulting in up to a 41% increase in terpene concentration (Reichel et al., 2024). Additionally, the light spectrum can influence plant morphology, which in turn affects the optimal plant density. For example, green light, due to its lower absorbance can penetrate deeper into the canopy and has been shown to enhance growth, yield, and secondary metabolite production, especially under high light intensities (Kim et al., 2004; Brodersen and Vogelmann, 2010).

The role of environmental factors is equally pivotal in modulating secondary metabolism. Climate change and ecological disturbances such as temperature fluctuations (Shohael et al., 2006; Nguyen et al., 2020), pH (Ncise et al., 2021), light quality (Paradiso and Proietti, 2022), nutrient availability (Olfati et al., 2012), elevated CO_2_ levels (Ziska et al., 2008; Ghasemzadeh et al., 2010), ozone exposure (Pellegrini et al., 2015), UV radiation and drought stress (Amirjani, 2013) induce significant physiological and biochemical responses in plants, often triggering the upregulation of secondary metabolite biosynthesis. These responses are often species-specific and exposure-dependent, with certain stressors increasing bioactive metabolite levels by up to 50%. For example, drought stress has been linked to elevated production of camptothecin in *Camptotheca acuminata* Decne (Nyssaceae) (happy tree) and withanolides in *Withania somnifera* L. (Solanaceae) (winter cherry) (Singh et al., 2017; Arya et al., 2025; Chawla et al., 2025). Such highlights how controlled stress exposure can be leveraged to enhance the medicinal value of plant-derived products.

We compiled a comparative table that outlines various strategies used to enhance secondary metabolites accumulation in medicinal plants, including mutation breeding, metabolic engineering, omics-guided selection, plant-microbe interactions, and environmental modulation (Table 5). Each strategy is evaluated based on multiple criteria such as efficacy, yield potential, cost, time requirements, purification complexity, scalability, precision, and regulatory challenges. This table serves as a practical decision-making tool for researchers seeking to select the most appropriate and efficient method for a given context. By providing a side-by-side comparison, it helps identify strategies that are both technically and economically viable, particularly for enhancing metabolites with immunomodulatory potential. Ultimately, it supports the design of targeted research and development efforts aimed at producing plant-based immune therapeutics in a sustainable and scalable manner.

**TABLE 5:** Comparative table summarizing different strategies for enhancing secondary metabolites production in medicinal plants.

Strategy	Efficacy	Yield potential	Cost	Time required	Purification complexity	Scalability	Precision	Regulatory barriers
Conventional breeding	Medium	Moderate	Low	Long	Moderate	High	Low	Low
Marker-Assisted Selection	High	High	Moderate	Moderate	Moderate	High	High	Moderate
Transgenic approaches	High	Very high	High	Long	High	Moderate	High	Very high
CRISPR/Cas genomic editing	Very high	Very high	Very high	Moderate	High	Low-moderate	Very high	High
Biostimulant application	Medium	Moderate	Low	Short	Low	High	Moderate	Low
Elicitor treatment	High	High	Low	Short	Low	High	Moderate	Low
*In vitro* cultures	High	High	High	Moderate-long	High	Moderate	High	Moderate
Mutation breeding	Medium	Variable	Moderate	Long	Moderate	Moderate	Low-Moderate	Low
Metabolic engineering	Very high	Very High	Very high	Long	High	Low	Very High	Very high
Omics-guided selection	High	High	High	Moderate-long	Moderate	Moderate	High	Moderate
Plant-microbe interactions	High	High	Low-moderate	Moderate	Low	High	Moderate	Low
Environmental modulation	Moderate	Moderate	Low	Short-Medium	Low	High	Low-Moderate	Low

### 5.1 Synthesis of Section 5

Collectively, the evidence presented in Section 5 highlights that agronomic practices, environmental modulation, and post-harvest treatments function as indirect yet highly influential drivers of secondary metabolite accumulation through controlled stress induction and metabolic reprogramming. A consistent pattern is that moderate, well-regulated stress conditions such as nutrient limitation, light manipulation, and drought stimulate key biosynthetic pathways. Particularly those associated with alkaloids, phenolics, and terpenoids, thereby enhancing the immunomodulatory potential of medicinal plants. However, these responses are highly context-dependent, with outcomes varying according to species, stress intensity, duration, and developmental stage, which limits reproducibility and predictability across production systems. This comparative inconsistency highlights a major limitation in current literature, where environmental enhancement strategies are frequently evaluated under controlled experimental conditions but insufficiently validated under scalable production systems.

Importantly, unlike genetic or elicitor-based strategies, agronomic and environmental approaches offer high scalability and low-cost implementation but lack precision in targeting specific metabolic pathways. This reflects a fundamental trade-off between practicality and control. The comparative analysis in Table 5 further reinforces that while advanced strategies such as CRISPR/Cas and metabolic engineering provide superior precision and yield potential, agronomic and environmental interventions remain more accessible and immediately applicable, particularly in low-resource settings.

Nevertheless, a critical limitation across these approaches is that enhanced metabolite accumulation does not inherently ensure improved immunomodulatory efficacy, as post-synthetic factors such as metabolite stability, extraction efficiency, and bioavailability remain significant constraints. Therefore, optimising immunomodulatory outcomes requires an integrated framework that combines controlled stress application with precise genetic and biochemical strategies, alongside optimised post-harvest processing. This integrative perspective advances current approaches by positioning agronomic and environmental modulation not merely as supportive practices, but as key components of a multi-layered strategy linking plant stress physiology, metabolic enhancement, and functional therapeutic potential.

## 6 Immunomodulatory mechanisms and therapeutic potential of major plant secondary metabolites

Rather than acting through a single pathway, metabolites exert multi-target immunomodulatory effects by influencing receptor sensitivity, cytokine production, antigen presentation, and immune cell differentiation (Sharma et al., 2017; Shen et al., 2023; Zahra et al., 2024). For example, alkaloids exhibit immunomodulatory activity through selective inhibition of immune cell activation and proliferation, particularly in T lymphocytes. Shen et al. (2023) demonstrated that alkaloids regulate major signalling cascades, including TNF-α/NF-κB, IL-6/JAK-STAT, IFN-γ/JAK/STAT, and IL-1/NF-κB pathways, which are critically involved in cytokine storm and excessive immune activation. These alkaloids were shown to exert multi-target immunomodulatory effects by reducing pro-inflammatory cytokine production, modulating immune-cell signalling, and restoring immune homeostasis in both *in vitro* and *in vivo* models.

Tetrandrine reduce T-cell responsiveness by limiting activation marker expression and cytokine production, thereby suppressing the clonal expansion of activated immune cells (Lai et al., 1999; Wang et al., 2018). Wang et al. (2018) demonstrated that tetrandrine significantly reduced IL-22-induced proliferation in HaCaT cells and downregulated pro-inflammatory cytokines including TNF-α, IL-1β, and IL-6. This effect was associated with inhibition of STAT3 phosphorylation, a central signalling node in inflammatory and immune responses.

Further, terpenoids exhibit immunomodulatory potential that is strongly associated with selective targeting of immune receptors and specific immune cell subsets. Certain terpenoids can interfere with receptor complex formation at the cell surface, thereby modulating immune activation thresholds and downstream signalling events (Sun et al., 2021). Ali et al. (2025) demonstrated through *in silico* analyses that specific terpenoid compounds could modulate the IFI16-AIM2 interaction, a regulatory axis associated with immune activation, tumour progression, and inflammatory responses. The study further showed that certain terpenoids exhibited strong binding affinity toward IFI16 and AIM2 proteins and were associated with enhanced immune-cell infiltration, particularly CD8^+^ T cells and dendritic cells, in lung squamous cell carcinoma models.

In addition, saponins exhibit strong immunostimulatory properties through direct interactions with cell membranes and immune receptors that enhance antigen uptake and presentation. For instance, QS-21 (plant-derived triterpenoid saponin adjuvant) promotes both humoral and cellular immunity by improving communication between antigen-presenting cells and lymphocytes, resulting in enhanced antibody production, immune memory formation, and cytotoxic T-cell responses (Talukdar et al., 2025). Mechanistically, den Brok et al. (2016) demonstrated that saponin-based adjuvants stimulate intracellular lipid body formation in dendritic cells, thereby enhancing antigen cross-presentation and subsequent T-cell activation. The study further showed that lipid body induction was directly associated with improved cellular immunity, highlighting the important role of saponins in regulating dendritic-cell-mediated immune responses.

Plant polysaccharides have received increasing attention due to their relatively low toxicity and broad immunological activity. Wang X.-F. et al. (2022) demonstrated that polysaccharides derived from medicinal plants such as *Aloe vera* (L.) Burm.f. (Asphodelaceae) (aloe vera), *Panax ginseng* C.A.Mey. (Araliaceae) (oriental ginseng), and *Lycium barbarum* L. (Solanaceae) (wolfberry)*,* regulate immune and inflammatory responses through key signalling pathways including NF-κB, PI3K/AKT, JNK, and TLR4. These findings support the concept that structurally complex plant-derived polysaccharides can modulate both innate and adaptive immunity through receptor-mediated mechanisms.

It is important to emphasise that most immunomodulatory evidence for secondary remains derived from *in vitro* or animal-based systems, with limited clinical validation. Furthermore, most studies focus on isolated metabolites or crude extracts, without adequately considering how agronomic or biotechnological enhancement of secondary metabolite accumulation may alter immunological outcomes. Changes in metabolite composition, concentration, and synergistic interactions may significantly influence receptor targeting and immune efficacy, yet these effects remain insufficiently characterised.

## 7 Discussion

This review is subject to several limitations, including variability in experimental methodologies across studies, and reliance on reported metabolite increases without standardized measurement protocols. Additionally, many studies infer immunomodulatory effects based on metabolite presence rather than directly demonstrating these effects in clinical or *in vivo* human systems. Future studies should aim to establish direct experimental links between enhanced metabolite accumulation and immunomodulatory effects in human or animal models, standardize analytical methods for measuring secondary metabolite levels, investigate pharmacokinetics, bioavailability, and metabolism of plant-derived metabolites, integrate multi-omics and systems biology approaches to better understand regulatory networks controlling secondary metabolism.

## 8 Conclusion

Enhancing the accumulation of secondary metabolites in medicinal plants is emerging as a powerful strategy to unlock and amplify their immunomodulatory potential. Secondary metabolites are not only central to plant defence but also possess profound pharmacological properties that modulate human immune functions. This review highlights a range of strategies from biostimulant and elicitor applications to genetic and environmental interventions that can be harnessed to increase the biosynthesis of these bioactive metabolites. Techniques such as foliar spraying with biostimulants, *in vitro* elicitation, traditional and transgenic breeding, and the use of beneficial microbes all play a critical role in triggering defence-related biosynthetic pathways.

Despite substantial progress in enhancing metabolite accumulation, a critical gap remains in establishing direct and mechanistic links between increased phytochemical production and consistent immunomodulatory efficacy in humans. In particular, the complexity of metabolic networks, variability in bioavailability, and post-synthetic modifications limit the translation of *in planta* gains into functional therapeutic outcomes. Future research should prioritise integrative, multi-omics approaches including genomics, transcriptomics, metabolomics, and proteomics to elucidate the regulatory networks governing secondary metabolite biosynthesis and their downstream biological effects. Future studies should further explore the enhancement of plant polysaccharides and other immunomodulatory metabolites through integrated biostimulant, elicitation, and genetic approaches, while also addressing challenges related to bioavailability, structural complexity, and clinical translation. Coupling these approaches with systems biology frameworks will enable the modelling of metabolic flux, pathway interactions, and genotype-environment-management interactions, thereby improving predictability and optimisation of metabolite production. This review therefore advances current literature by moving beyond descriptive assessments of metabolite enhancement strategies toward a more mechanistic and translational interpretation of how metabolic reprogramming influences therapeutic applicability. Furthermore, translational research frameworks that link plant-derived metabolites to clinical and pharmacokinetic outcomes are essential to validate their immunomodulatory potential under real-world conditions. Ultimately, integrating biostimulant, elicitor, genetic, and agronomic strategies within a systems-level and translational framework offers a scalable, precise, and sustainable pathway for developing next-generation plant-based immunomodulators capable of addressing emerging global health challenges.
